# Loss of the Mia40a oxidoreductase leads to hepato-pancreatic insufficiency in zebrafish

**DOI:** 10.1371/journal.pgen.1007743

**Published:** 2018-11-20

**Authors:** Anna M. Sokol, Barbara Uszczynska-Ratajczak, Michelle M. Collins, Michal Bazala, Ulrike Topf, Pia R. Lundegaard, Sreedevi Sugunan, Stefan Guenther, Carsten Kuenne, Johannes Graumann, Sherine S. L. Chan, Didier Y. R. Stainier, Agnieszka Chacinska

**Affiliations:** 1 International Institute of Molecular and Cell Biology, Warsaw, Poland; 2 Department of Developmental Genetics, Max Planck Institute for Heart and Lung Research, Bad Nauheim, Germany; 3 Biomolecular Mass Spectrometry, Max Planck Institute for Heart and Lung Research, Bad Nauheim, Germany; 4 Centre of New Technologies, University of Warsaw, Warsaw, Poland; 5 Department of Biomedical Sciences, Faculty of Health and Medical Sciences, University of Copenhagen, Copenhagen, Denmark; 6 Bioinformatics and Deep Sequencing Platform, Max Planck Institute for Heart and Lung Research, Bad Nauheim, Germany; 7 Department of Drug Discovery and Biomedical Sciences, College of Pharmacy, Medical University of South Carolina, Charleston, South Carolina, United States of America; University of Cologne, GERMANY

## Abstract

Development and function of tissues and organs are powered by the activity of mitochondria. In humans, inherited genetic mutations that lead to progressive mitochondrial pathology often manifest during infancy and can lead to death, reflecting the indispensable nature of mitochondrial biogenesis and function. Here, we describe a zebrafish mutant for the gene *mia40a (chchd4a)*, the life-essential homologue of the evolutionarily conserved Mia40 oxidoreductase which drives the biogenesis of cysteine-rich mitochondrial proteins. We report that *mia40a* mutant animals undergo progressive cellular respiration defects and develop enlarged mitochondria in skeletal muscles before their ultimate death at the larval stage. We generated a deep transcriptomic and proteomic resource that allowed us to identify abnormalities in the development and physiology of endodermal organs, in particular the liver and pancreas. We identify the acinar cells of the exocrine pancreas to be severely affected by mutations in the MIA pathway. Our data contribute to a better understanding of the molecular, cellular and organismal effects of mitochondrial deficiency, important for the accurate diagnosis and future treatment strategies of mitochondrial diseases.

## Author summary

Mitochondrial pathologies which result from mutations in the nuclear DNA remain incurable and often lead to death. As mitochondria play various roles in cellular and tissue-specific contexts, the symptoms of mitochondrial pathologies can differ between patients. Thus, diagnosis and treatment of mitochondrial disorders remain challenging. To enhance this, the generation of new models that explore and define the consequences of mitochondria insufficiencies is of central importance. Here, we present a *mia40a* zebrafish mutant as a model for mitochondrial dysfunction, caused by an imbalance in mitochondrial protein biogenesis. This mutant shares characteristics with existing reports on mitochondria dysfunction, and has led us to identify novel phenotypes such as enlarged mitochondrial clusters in skeletal muscles. In addition, our transcriptomics and proteomics data contribute important findings to the existing knowledge on how faulty mitochondria impinge on vertebrate development in molecular, tissue and organ specific contexts.

## Introduction

Mitochondria are important organelles with multiple cellular functions, serving as energy- and biosynthetic centres, participating in Ca^2+^ signalling and cell stress responses, and executing cell death. Thus, compromised mitochondrial activity is associated with numerous human pathologies. A group of genetically inherited diseases that are relatively rare but present devastating symptoms include encephalomyopathies, Leber’s Hereditary Optic Neuropathy (LHON), Leigh Syndrome and Barth Syndrome [1–3]. Another group of pathologies relate to more common, age-related and genetically predisposed conditions. These are often associated with neurodegeneration, such as Parkinson’s and Alzheimer’s disease, or Amyotrophic Lateral Sclerosis (ALS) [4–6]. Finally, given that mitochondria orchestrate cellular metabolism, pathological conditions with symptoms of altered metabolism, including diabetes, obesity, cancer, as well as aging processes, have also been associated with mitochondrial dysfunction [7, 8]. Tissues with high energy demands such as the heart, brain, muscles and liver are typically most affected in patients with mitochondrial disease [1–3]. The fact that treatment of mitochondrial disorders is mostly directed at mitigating their symptoms reflects our relatively poor understanding of the biology of these disorders. It also underlines the necessity to generate new tools and models to study the molecular, cellular and organ related consequences of mitochondrial malfunction in complex organisms.

The mitochondrial proteome is of dual origin. While a handful of proteins are encoded by mitochondrial DNA (mtDNA), the vast majority are encoded by nuclear DNA. Thus, genetically determined mitochondrial diseases are heterogeneous and can be the consequence of mutations in either genome [1, 2, 7–9]. Nuclear-encoded mitochondrial proteins are synthesised on cytosolic ribosomes. Efficient import of such precursor proteins from the cytosol into their destined mitochondrial sub-compartments is critical to maintain the biogenesis of the complex organelle and attributed to the interplay of mitochondrial translocases [10–13]. The vast majority of our knowledge on the mechanism and function of the evolutionarily conserved import machineries stems from studies in unicellular organisms, such as the yeast *S*. *cerevisiae* [11]. However, the effect of impaired mitochondrial protein biogenesis on the development of vertebrates remains largely unexplored.

Mitochondrial translocases sort precursor proteins according to signals harboured within the polypeptide sequence. The import and maturation of cysteine-rich mitochondrial precursor proteins relies on the Mitochondrial Intermembrane space and Assembly (MIA) pathway. Mia40 (Chchd4), a central oxidoreductase of the mitochondrial intermembrane space, drives these final steps of the biogenesis of cysteine-rich mitochondrial proteins [11, 14–17]. The oxidation-coupled import of MIA substrates leaves Mia40 at a reduced state which is regenerated by its partner Augmenter of Liver Regeneration (Erv1/ALR) [18, 19]. Importantly, the majority of Mia40 substrates are directly or indirectly involved in the assembly of the respiratory complexes, which are often disturbed in pathological conditions [14, 20–22].

Animal models have historically been a source of knowledge on the mechanisms of a plethora of human pathologies. In zebrafish, mitochondrial dysfunction has been predominantly modelled by anti-sense approaches, transiently mimicking a deficiency of mitochondrial protein associated with a human disease [23–29]. In addition, defects in mitochondrial proteins were found to underlie specific phenotypes identified in forward genetic screens. Studies on the *oliver* mutant identified Tomm22, a component of the translocase of the outer mitochondrial membrane, to be essential for hepatocyte survival and consequently for the development of the liver in zebrafish [30]. *Xavier* and *dark xavier* zebrafish mutants carry mutations in genes encoding the electron transfer flavoprotein dehydrogenase (*etfdh*) [31] and the electron transfer flavoprotein a (*etfa*) [32], respectively. These mutations trigger phenotypic abnormalities similar to those observed in the human Multiple Acyl-CoA Dehydrogenase Deficiency syndrome (MADD), proving the zebrafish to be a valid model to study the highly evolutionarily conserved processes that operate in mitochondria. A study on mtDNA deficiency, modelled in zebrafish by introducing mutations in the gene that encodes the mtDNA polymerase (DNA polymerase gamma, *polg*), shows that mutant larvae were able to survive to the juvenile stage despite a progressive decrease in the level of mtDNA [33]. The biochemical and morphological phenotype of early onset Parkinson disease was recapitulated in zebrafish by introducing mutations in the PTEN-induced kinase-encoding gene (*pink1*) [34, 35], further supporting the use of zebrafish to model human pathologies.

The MIA pathway has been previously modulated in zebrafish by targeting the Mia40 partner Erv1/ALR. Using anti-sense technology, it was reported that Erv1/ALR plays an important role in the development of the liver and the pancreas [36]. Another approach using a chemical inhibitor of Erv1/ALR and a translation-blocking morpholino suggested a role during heart development [37]. However, with the recent advancement in genome engineering techniques, phenotypic discrepancies between knockdown and knockout are frequently reported in zebrafish [38, 39]. To date, no study investigated how mutations in genes encoding MIA pathway components impinge on the development of vertebrates. In this study, we used Transcription Activator-Like Effector Nucleases (TALENs) to generate *mia40* mutants in zebrafish to model mitochondrial dysfunction caused by an imbalance in mitochondrial protein biogenesis. We first characterised the two paralogues of *mia40* in zebrafish, *mia40a* and *mia40b*. We showed that both paralogues exhibit Mia40 activity, as they both rescued the lethal phenotype of *S*. *cerevisiae* devoid of endogenous *MIA40*. By generating mutations in both paralogues, we also found that *mia40a*, but not *mia40b*, is essential for survival in zebrafish. At the cellular level, genetic disruption of the MIA pathway led to abnormal, enlarged mitochondrial structures in skeletal muscles. At the organismal level, the resulting mitochondrial deficiency triggered a glycolytic phenotype and ultimately starvation. Global transcriptomics and proteomics analyses indicated abnormal development of the liver and the pancreas in *mia40a* mutants. Taken together, our findings contribute to the understanding of the consequences of compromised mitochondrial biogenesis at the organismal level.

## Results

### The MIA pathway is conserved in zebrafish

**Fig 1:**
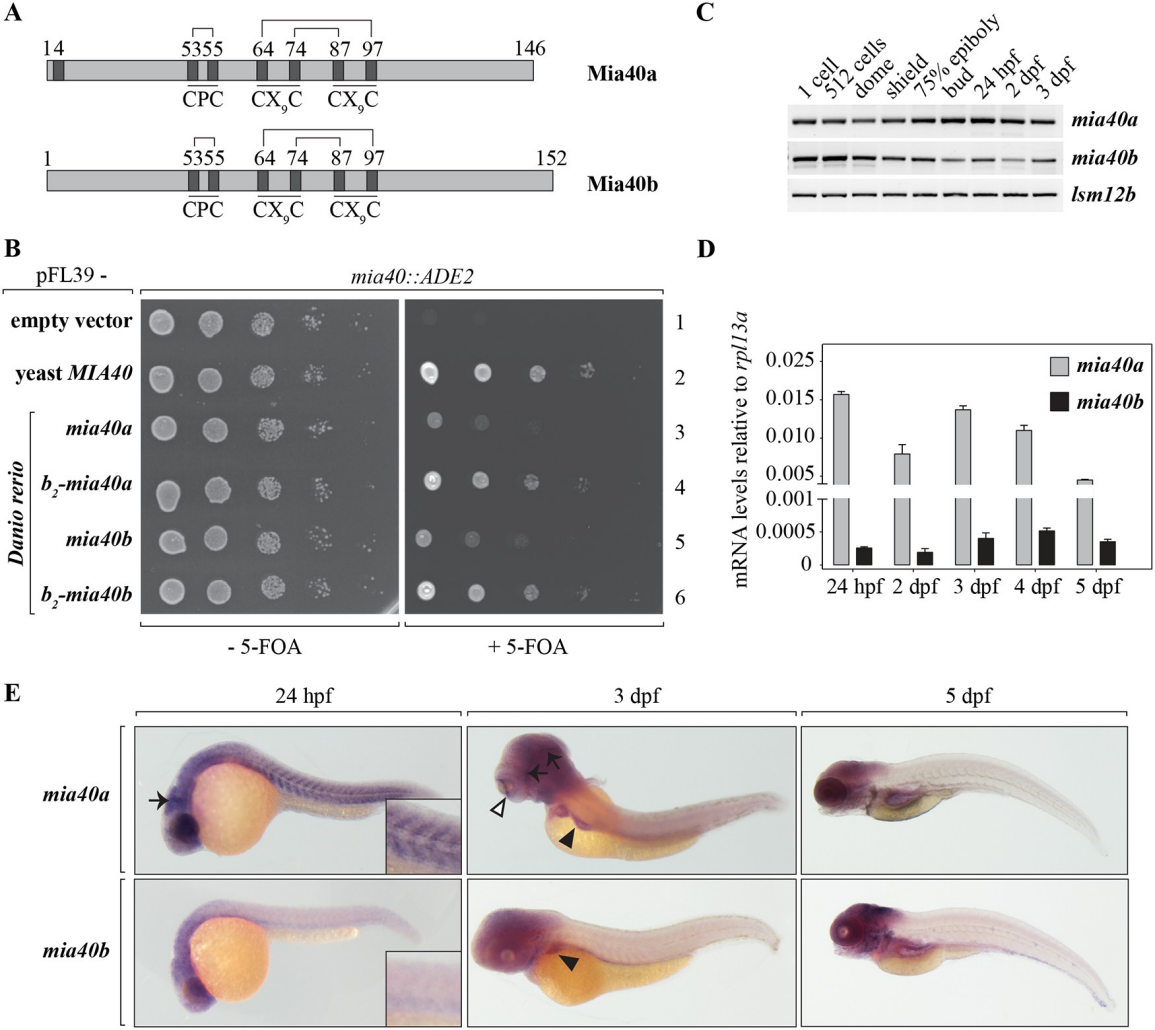
Two functional paralogues of *mia40* are present in the zebrafish genome. (A) Schematics of the protein products of the two paralogues of *mia40* in zebrafish. Disulfide bonds between conserved cysteines are marked. (B) The two zebrafish paralogues were expressed in yeast strains devoid of *MIA40*. The lethal phenotype of yeast strains depleted of *MIA40* is rescued by the expression of *mia40a* or *mia40b*. Growth is restored to levels of native yeast Mia40 upon fusion of *Danio rerio* paralogues to the *b2*-mitochondria targeting presequence. (C-E) The spatiotemporal expression of *mia40* paralogues in zebrafish development was monitored by RT-PCR (C), RT-qPCR (D) and *in situ* hybridization (E). (C) Transcripts for both *mia40* paralogues are maternally contributed as well as expressed by the embryo during early zebrafish development. (D) Relative to *rpl13a* expression, *mia40a* mRNA levels are significantly higher compared to the levels of *mia40b*. Error bars correspond to SEM; C_T_ values are presented in S8 Table. (E) mRNA expression patterns of *mia40a* and *mia40b* by *in situ* hybridization at indicated time points. At 24 hpf, *mia40a* expression is detected in the eye, brain (arrow), and somites, while *mia40b* expression is detected only in the head. Insets show a higher magnification of the somites. At 3 dpf, *mia40a* expression is enriched in the eye (white arrowhead) and brain (black arrows), and both *mia40a* and *mia40b* are expressed in the liver (black arrowheads). *mia40a* expression is greatly reduced by 5 dpf, whereas *mia40b* expression is strong in the head, branchial arches, epidermis, and lateral line primordia.

We investigated the role of mitochondrial biogenesis in vertebrate development by compromising the MIA pathway in *Danio rerio*. First, we focused on characterising the zebrafish Mia40 protein. As a consequence of a teleost-specific genome duplication [40], the zebrafish genome contains two copies of the *mia40 (chchd4)* gene, referred to as *mia40a (chchd4a)* and *mia40b (chchd4b)*. When comparing the protein sequences of Mia40a and Mia40b with the human, mouse, frog and yeast orthologues (S1A Fig) it is apparent that both zebrafish protein paralogues retained the conserved cysteine motifs, the redox-active cysteine-proline-cysteine (CPC) and the twin CX_9_C motifs (Fig 1A). These motifs underlie the enzymatic activity of Mia40 and its intermembrane-space import and retention, respectively (Fig 1A and S1A Fig). Nonetheless, the protein sequence alignment (S1A Fig) and the phylogenetic analysis (S1B Fig) reveal significant differences between the two zebrafish paralogues, including amino-acid residue identity of less than 50%.

To determine whether the two proteins are enzymatically active, we performed a yeast complementation assay. The two zebrafish paralogues were expressed separately in yeast *S*. *cerevisiae* deleted for *MIA40*, under the endogenous promoter of yeast *MIA40*. We observed that expression of *mia40a* or *mia40b* partially rescued the lethal phenotype of the loss of Mia40 in yeast (Fig 1B, compare right panel lane 1 with 3 and 5). In contrast to its homologues in higher eukaryotes, yeast Mia40 is not a soluble protein in the intermembrane space. Due to an N-terminally located presequence and transmembrane domain, it is instead anchored in the inner mitochondrial membrane (S1A Fig) [41]. To faithfully mimic the import and maturation of yeast Mia40, we fused the zebrafish *mia40a* or *mia40b* with the cytochrome *b*_2_ bipartite signal sequence and expressed it in Mia40-deficient yeast [41]. We observed that both fusion proteins were able to sustain yeast viability at a similar level to that of the endogenous protein (Fig 1B, compare right panel lane 2 with 4 and 6). Thus, we conclude that both zebrafish paralogues, *mia40a* and *mia40b*, exhibit Mia40 activity.

To characterise the function of the *mia40* genes in zebrafish, we first examined their spatiotemporal expression pattern. We performed RT-PCR analysis on RNA isolated from zebrafish embryos at various developmental stages, from the zygote to 3 days post fertilization (dpf). We found that mRNA for both paralogues was maternally contributed and expression persisted until at least 3 dpf (Fig 1C). Next, we compared the expression of *mia40a* and *mia40b* mRNA to the level of a known, abundant mRNA of the housekeeping gene encoding the 60S ribosomal protein L13a (*rpl13a*) by quantitative PCR (RT-qPCR). While the expression of both zebrafish paralogues was lower compared to the reference *rpl13a*, *mia40a* transcript was more abundant compared to *mia40b* (Fig 1D).

We next sought to dissect the spatiotemporal distribution of *mia40a* and *mia40b* transcripts in zebrafish larvae by *in situ* hybridization with transcript-specific RNA probes. We observed that *mia40a* was strongly expressed in organs of high-energy demand including somites, brain and the eye at 24 hours post fertilization (hpf) (Fig 1E). In contrast, *mia40b* transcripts were expressed in the head and eye, but absent from developing somites at 24 hpf (Fig 1E). At 3 dpf, both transcripts were expressed in the liver and eye. Although *mia40a* expression was strongly reduced at later time points, we conclude that its expression was generally broader compared to that of *mia40b*. At 5 dpf, *mia40b* expression was detected in the head, branchial arches, epidermis, and lateral line primordia (Fig 1E).

**Fig 2:**
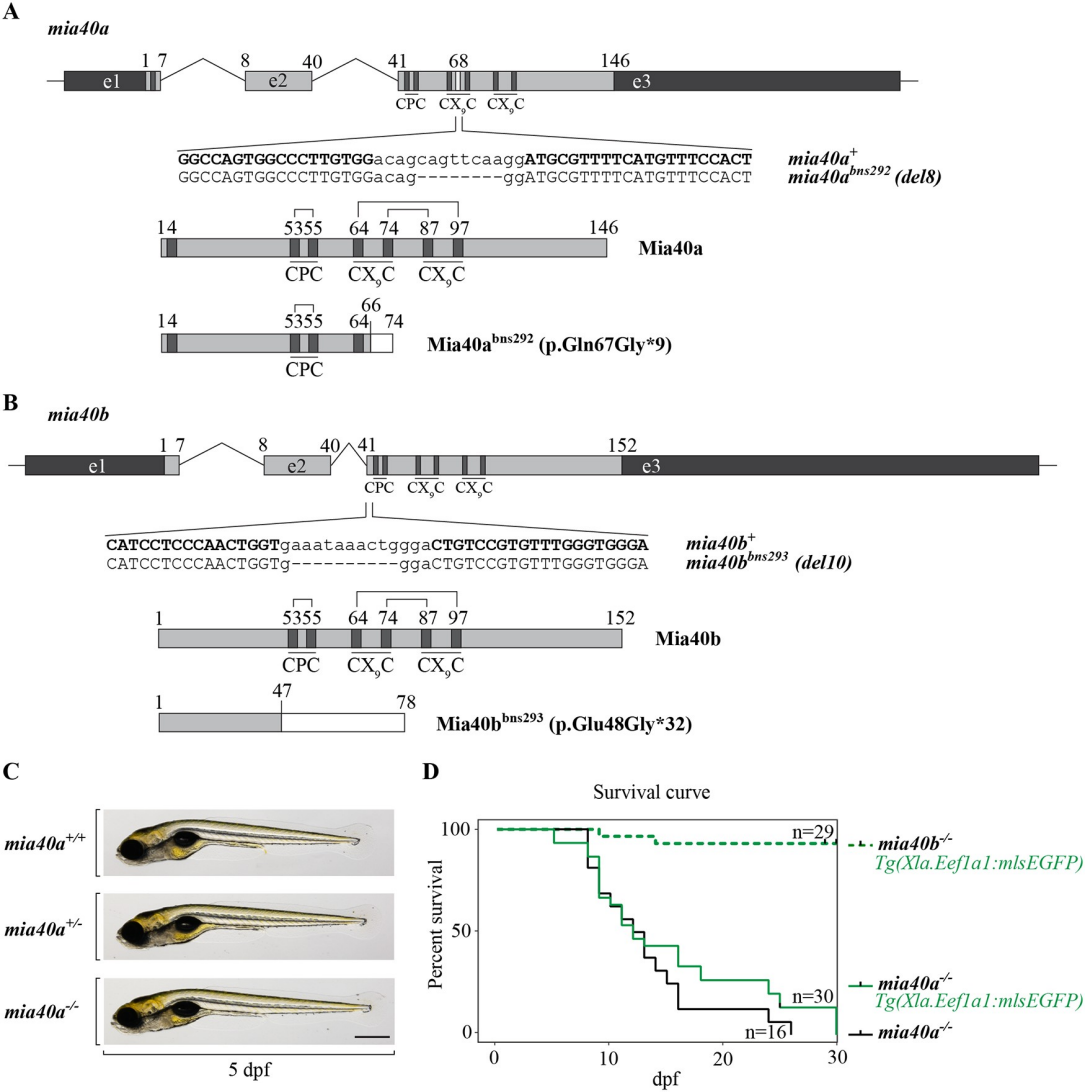
Mia40a is essential for survival in zebrafish. TALENs were used to obtain *mia40a* and *mia40b* mutants (A, B). (A) A TALEN was designed to target the locus encoding Phe68 of *mia40a*. The identified 8 base pair (bp) deletion and the predicted protein product for the *mia40a*^*bns292*^ allele are shown (bottom panels). In bold are TALEN arm-recognized DNA sequences. (B) A TALEN targeting the CPC-encoding region of *mia40b* was used to obtain a mutant with a truncated form of Mia40b. The identified 10 bp deletion leads to a frameshift mutation with a premature stop codon. The predicted protein product for the *mia40b*^*bns293*^ allele is shown (bottom panels). In bold are TALEN arm-recognized DNA sequences. (C) *mia40a* homozygous mutants do not present any gross morphological defects compared to their siblings at 5 dpf. Scale bar, 500 μm. (D) Homozygous *mia40a* mutants die during mid-larval to juvenile developmental stages while *mia40b* mutants survive to adulthood and give rise to progeny. The results are derived from AB or *Tg(Xla*.*Eef1a1*:*mlsEGFP)* genetic background. n: number of analysed individuals.

To evaluate the loss of Mia40 function *in vivo*, we used TALEN gene editing to generate zebrafish lines with mutations in the two *mia40* paralogues. The third exon of *mia40a* was targeted for mutagenesis (Fig 2A). A TALEN was designed to target the sequence encoding the conserved phenylalanine 68, located on the hydrophobic concave surface that supports Mia40 interaction with its substrates (Fig 2A) [42–44]. We recovered a delta 8 (*bns292*) indel in *mia40a*. The predicted protein product in *mia40a*^*bns292*^ mutants (also referred to as *mia40a*^*-/-*^ or *mia40a* mutants) contains a frameshift mutation in the 67th codon leading to a premature stop codon (p. Gln67Gly*9) (Fig 2A). In parallel, the third exon of *mia40b* paralogue was TALEN- targeted. A delta10 (*bns293*) indel was recovered, preceding the CPC-coding region of *mia40b*. The predicted protein product in *mia40b*^*bns293*^ mutants (also referred to as *mia40b*^*-/-*^ or *mia40b* mutants) contains a frameshift mutation and yields a truncated protein product (p.Glu48Gly*32) (Fig 2B).

By in-crossing the *mia40a*^*+/bns292*^ or *mia40b*^*+/bns293*^ zebrafish, we observed no severe developmental abnormalities among siblings up to 5 dpf (*mia40a*: Fig 2C). However, while *mia40b*^*-/-*^ larvae survive to adulthood and are fertile, no individuals homozygous for the mutation in *mia40a* are recovered. To monitor in detail the survival of homozygous mutants of *mia40a* or *mia40b*, we genotyped the larvae at 72 hpf by fin-fold amputation, separated larvae according to genotype and monitored their growth. We noticed that in contrast to *mia40b* homozygous mutants, *mia40a*^*-/-*^ larvae succumb to death starting at 10 dpf, reaching 50% lethality by 2 weeks of age (Fig 2D). The observation that *mia40b*^-/-^ show no growth retardation and that wild-type Mia40b protein is not able to compensate for the loss of Mia40a in *mia40a* mutants provides evidence for non-redundant roles of these paralogues in zebrafish.

### *mia40a* mutants present altered mitochondrial structures in skeletal muscle

**Fig 3:**
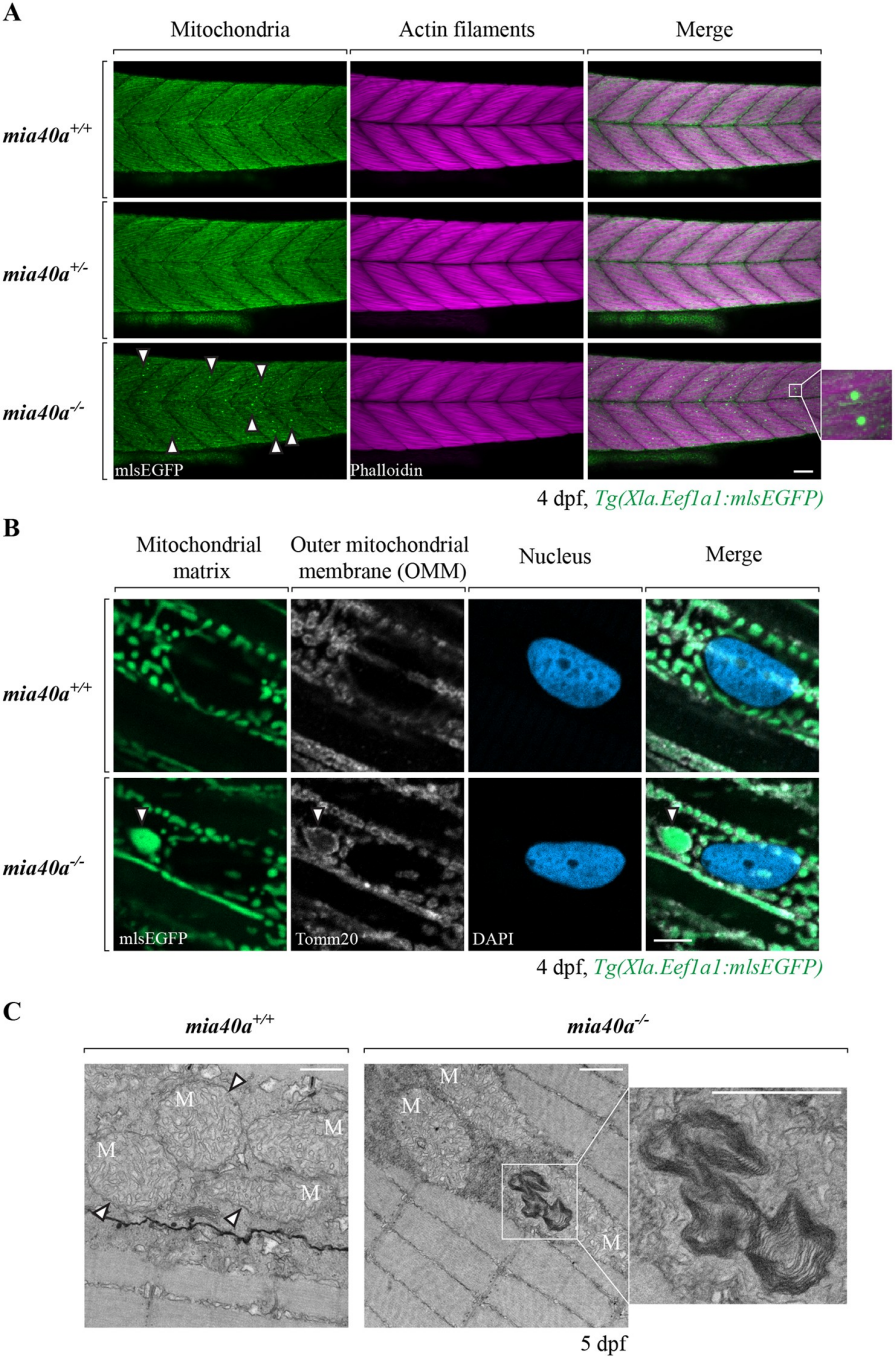
Abnormal mitochondrial structures are found in the skeletal muscles of *mia40a* mutants. (A) Embryos obtained from an in-cross of heterozygous *mia40a*^*+/-*^ siblings in the *Tg(Xla*.*Eef1a1*:*mlsEGFP*) background were stained with phalloidin to visualize actin filaments of the skeletal muscle at 4 dpf. Abnormal, enlarged mitochondrial structures are found in the skeletal muscles of the *mia40a* mutants (arrowheads). To better visualize the GFP-positive inclusions, a magnified image is shown on the side. Images are maximum projections. Scale bar, 50 μm. All images are lateral views, anterior to the left. (B) wild-type and *mia40a*^*-/-*^ larvae in the *Tg(Xla*.*Eef1a1*:*mlsEGFP*) background were stained with an anti-Tomm20 antibody to mark the outer mitochondrial membrane (OMM). DNA was counterstained with DAPI. Single plane sections are shown. The arrowhead points to a GFP-positive inclusion. Scale bar, 5 μm. All images are lateral views, anterior to the left. (C) wild-type or *mia40a*^*-/-*^ siblings in AB background were subjected to transmission electron microscopy (TEM) analysis at 5 dpf. Arrowheads point to well defined mitochondrial membranes in wild-type larvae. Abnormal membranous structures found in the *mia40a* mutants are magnified (inset). Scale bar, 500 nm. M: Mitochondria.

The skeletal muscles of zebrafish are rich in mitochondria [45, 46]. We used the zebrafish *Tg(Xla*.*Eef1a1*:*mlsEGFP)* line [47] to study mitochondrial morphology in the skeletal muscles of the *mia40a* mutants. This transgenic line expresses a mitochondrial matrix-targeted GFP under a ubiquitous promoter. We observe prominent, GFP-positive inclusions in skeletal muscle cells of the *mia40a*^*-/-*^ but not in *mia40a*^*+/+*^ or *mia40a*^*+/-*^ siblings (Fig 3A). Inclusion-positive mutant larvae do not show any abnormalities in the organisation of the actin filaments in the muscles, as visualized by phalloidin staining (Fig 3A). Interestingly we were able to rescue the inclusion phenotype at 3 dpf by injection of wild-type *mia40a* or *mia40b* mRNA at the 1-cell stage (S2A and S2B Fig, left panels). In contrast, the injection of mRNA encoding the mutant alleles had no effect on the presence of inclusions (S2A and S2B Fig, right panels).

The import of mitochondrial matrix-targeted precursor proteins requires an electrochemical membrane potential across the inner mitochondrial membrane. This potential is generated by functional electron transport chain (ETC). Therefore, we asked whether the GFP-positive inclusions are mitochondrial or cytosolic pools of GFP that fail to be imported into the mitochondrial matrix due to ETC insufficiency. Using an antibody against Tomm20, a component of the translocase of the outer membrane (TOM) complex, we observed the GFP signal to be surrounded by the outer membrane in commonly occurring mitochondria as well as GFP-positive, enlarged structures (Fig 3B, bottom panel, arrowhead). From these data, we conclude that *mia40a* deficiency results in large inclusions that represent malformed mitochondria.

Transmission electron microscopy (TEM) of 5 dpf old larvae revealed osmiophilic inclusions in the mitochondria of the skeletal muscles in the tail of *mia40a* mutant larvae (Fig 3C). The inclusions have a resemblance of myelin-like bodies (Fig 3C, inset). Apart from these inclusions, mutant mitochondria show well-conserved morphology in general, with well-defined cristae. Some mutant mitochondria present less well-compacted outer membrane compared to the wild-type siblings. Skeletal sarcomeres are well-structured and well-defined in *mia40a*^*-/-*^ larvae (Fig 3C), suggesting that muscle structure is intact, in line with conclusions derived from phalloidin staining experiments (Fig 3A).

### MIA insufficiency in zebrafish triggers a metabolic switch and starvation

**Fig 4:**
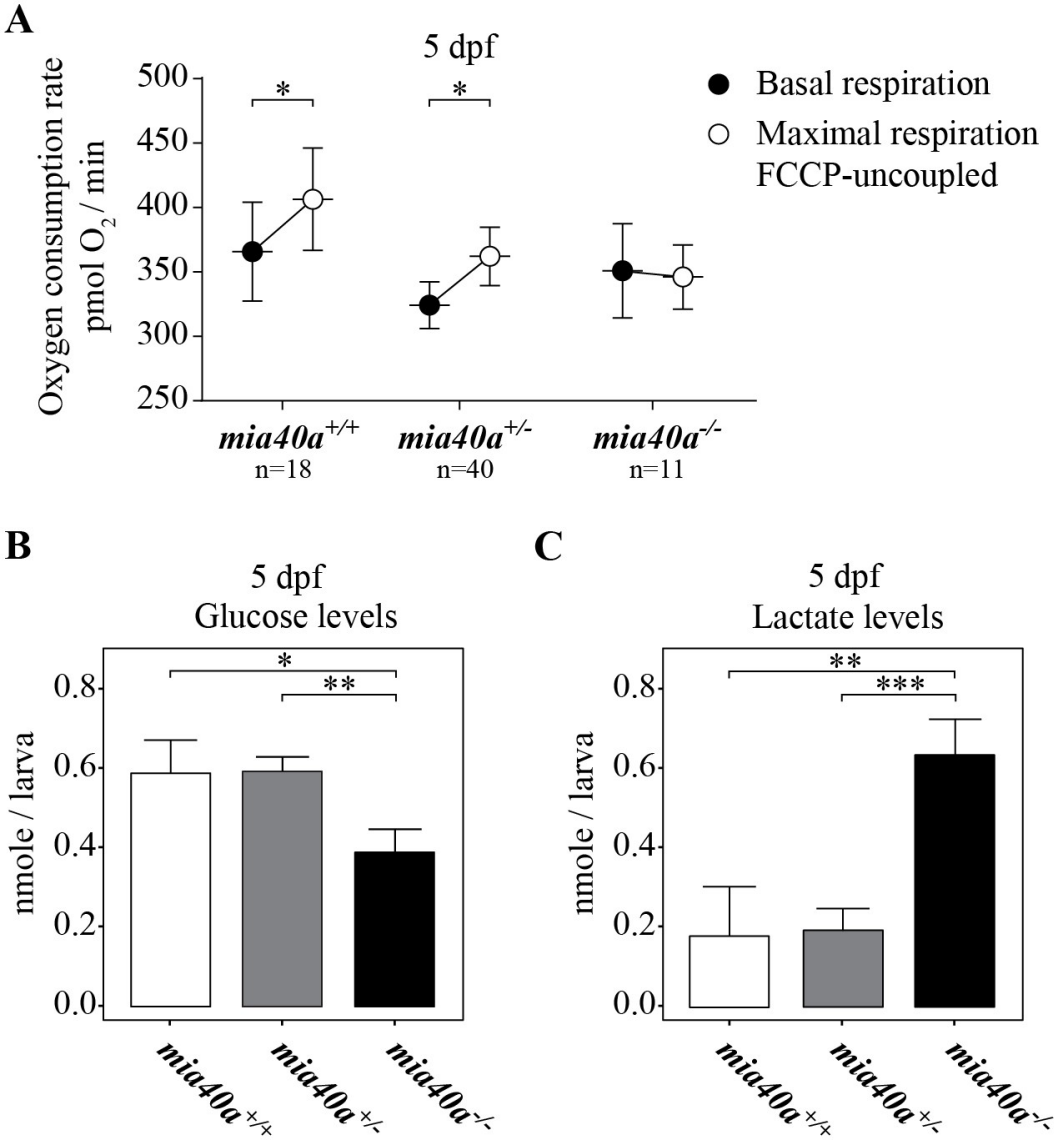
Mutations in *mia40a* trigger respiration defects and a metabolic shift. Embryos obtained from an in-cross of heterozygous *mia40a*^*+/-*^ siblings were collected and subjected to respiration analysis using the Seahorse Bioscience XF-24 extracellular flux analyzer (A) and metabolite analysis (B,C) at 5 dpf. (A) No significant changes in cellular respiration between the compared genotypes are observed at 5 dpf. However, *mia40a* mutant larvae respire at their maximal capacity as FCCP-treatment failed to trigger an increase in respiration. Error bars correspond to SEM; *P* < 0.05 (*) by Mann-Whitney test; n: number of analysed individuals. At 5 dpf, the *mia40a* mutants show a glycolytic phenotype marked by a decreased level of glucose (B) and an increase in lactate (C). Data derived from three (glucose) and four (lactate) biological replicates. Error bars correspond to SEM; *P* < 0.05 (*), *P* < 0.01 (**) and *P* < 0.001 (***) by unpaired *t*-test.

We next investigated mitochondrial function in *mia40a* mutants and measured respiration that reflects mitochondrial energetics *in vivo*. Interestingly, we found that MIA pathway insufficiency does not trigger changes in the basal oxygen consumption rate at 5 dpf (Fig 4A). Mitochondrial spare respiratory capacity (SRC) represents a measure for the ability to respond to an increased energy demand and is established by subtracting the basal respiration from maximal respiration. Maximal respiration can be measured upon uncoupling of the mitochondrial membrane potential using the FCCP ionophore. Importantly, unlike wild-type and heterozygous siblings, exposure to FCCP did not trigger an increase in respiration rate in *mia40a* mutants (Fig 4A). Taken together, these data suggest that *mia40a* mutants fully utilize mitochondrial capacity to meet respiratory demands of 5 dpf larvae with little or no spare capacity. Unexpectedly, we found that the relative level of mtDNA was increased in the *mia40a*^*-/-*^ larvae at this stage (S3A Fig).

The yolk of zebrafish larvae is nearly depleted at 5 dpf, which coincides with a peak in whole body glucose content in larvae that have never been fed. After 5 dpf, the larvae start fasting and glucose levels decrease unless exotrophic nutrition is introduced [48]. We analysed the whole-body glucose levels in 5 dpf larvae and found that *mia40a* mutants present a significant decrease in glucose content compared to wild-type and heterozygous siblings (Fig 4B). As the yolk size was similar between the genotypes at all time points analysed, we conclude that glucose is consumed as an alternative energy source via the glycolytic pathway. Indeed, at 5 dpf, the level of lactate, a by-product of glycolysis, is significantly increased in *mia40*^*-/-*^ larvae (Fig 4C), further supporting this conclusion.

We next investigated the metabolic state of mutant larvae at later time points. At 10 dpf, a drop in *mia40a* mutant survival was observed (Fig 2D). Measuring basal respiration in living larvae at 10 dpf, we found that *mia40a* mutants respire at levels similar to those of the starved wild-type and heterozygous siblings (S3B Fig). Upon introduction of exotrophic nutrition from 5 dpf onwards, *mia40a*^*+/+*^ and *mia40a*^*+/-*^ larvae significantly increase oxygen consumption (S3B Fig). In contrast, homozygous mutants retain respiration at an equal level to starved animals (S3B Fig). We thus conclude that post 5 dpf, when yolk-derived nutrition is exhausted, phenotypes in *mia40a* mutant larvae are likely consequences of starvation. As predicted from this conclusion, glucose levels are barely detected in the *mia40a* homozygous mutants at 10 dpf (S3C Fig).

Based on the observations that *mia40a* mutants show challenged cellular respiration already at 5 dpf and its progressive decrease later in the development, we conclude that they represent a valid model of mitochondrial insufficiency caused by compromised mitochondrial protein biogenesis. In support, we have observed that subunits of respiratory complex I (Ndufa9) and complex IV (Cox4i1) are reduced in *mia40a* mutants at 5 dpf (S4 Fig) compared to other mitochondrial proteins (Atp5a) and cytosolic controls (Rpl7, β Actin) in line with previous reports [20].

### Transcriptomic and proteomic changes triggered by the loss of Mia40a

**Fig 5:**
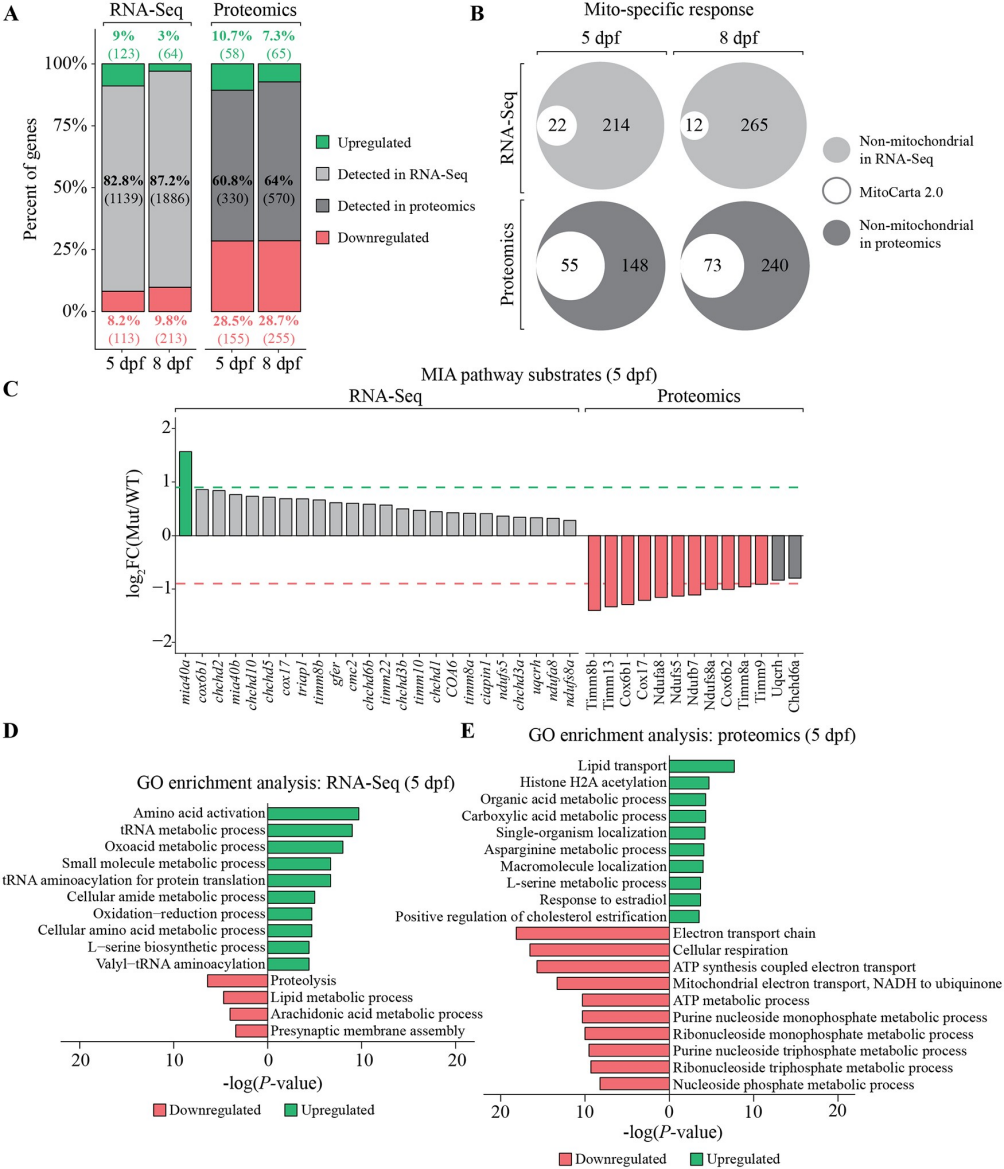
Global expression analysis at the transcriptomic and proteomic levels. Transcriptomic profiling and proteomic analysis of *mia40a* mutants (Mut) compared to wild type (WT) at 5 or 8 dpf. (A) Bar plots representing the total number of genes and proteins identified in 5 and 8 dpf samples by RNA sequencing (RNA-Seq) and quantitative proteomic analysis. The proportions of significant genes or proteins (FDR 5%) are shown in grey shades, while the proportions of differentially expressed features in the mutant samples (FDR 5%; log_2_FC = < -0.9 and > = 0.9) are shown in green (upregulated) and red (downregulated). The number of genes and proteins in each fraction are indicated in parenthesis. (B) Venn diagram representing the number of mitochondrial genes and proteins from the MitoCarta 2.0 repository in each set [94]. Mitochondrial genes and proteins are highlighted in white, whereas the remaining non-mitochondrial, differentially expressed features are presented in greys. This analysis was done on the Ensembl gene IDs level. (C) Expression analysis of Mia40a substrates in transcriptomic and proteomic data for 5 dpf samples is shown. The red dashed line represents log_2_FC = -0.9 and the green dashed line represents log_2_FC = 0.9. Gene ontology (GO) enrichment analysis for biological process of 236 and 213 differentially expressed genes (D) and proteins (E), respectively. The results are shown as a negative log_10_ *P*-value after Bonferroni correction. Bars in green indicate GO terms enriched for upregulated genes or proteins, while the red bars represent the GO terms for the downregulated features. For better representation of the results, the bars enriched for downregulated genes or proteins were transformed to be located on the left hand side of the plot.

To gain more insights into molecular processes and pathways affected, we determined global changes at the transcriptomic and proteomic levels by isolating total RNA and proteins at 5 and 8 dpf from *mia40a* mutants (Mut) and wild-type (WT) siblings. We used RNA sequencing (RNA-Seq) and quantitative proteomic analysis to identify differentially expressed genes and proteins between *mia40a* mutants and corresponding wild-type siblings. In total, the differential analysis showed expression changes of 236 genes and 213 proteins (203 unique genes) at 5 dpf and 277 genes and 320 proteins at 8 dpf (FDR 5%; log_2_FC = < -0.9 or > = 0.9) (Fig 5A, S1 Table). Generally, we observed that the proteomics approach yielded a higher proportion of differentially regulated and predominantly decreased features (in red, right bars, Fig 5A), hinting at a post-translational origin of the differences. We proceeded to identify what proportion of the differentially expressed genes is targeted to mitochondria. Similarly, proteomics yielded more mitochondrial features (up to 27% for 5 dpf) compared to transcriptome profiling (less than 10% for both time points), suggesting that the response triggered by the depletion of Mia40a is more pronounced at the proteomic rather than transcriptomic level (Fig 5B, S2 Table). These observations further strengthen the hypothesis that post-translational processes are likely involved in the mitochondrial phenotype. As the cellular milieu for Mia40a activity are mitochondria and Mia40a deficiency affects post-translational protein processing, proteomics yielded a higher percentage of mitochondrial proteins among those strongly affected in the mutant (Fig 5B, compare bottom panels to top panels). Due to the possible bias from starvation on the expression of genes and proteins in 8 dpf *mia40a* mutant larvae, further analysis focused on 5 dpf samples.

Next, we carefully investigated the expression changes for MIA pathway substrates [15, 21, 49–51] and noticed a more severe response at the proteomic level (Fig 5C). The proteins identified as substantially depleted, included several classical CX_3_C motif-containing chaperones of the intermembrane space (Timms), as well as important components of the respiratory chain, represented by such proteins as Cox6b, Ndufa8 or Ndufs5 (Fig 5C). Interestingly, the transcriptomic response reveals mild, but global, upregulation of the MIA pathway substrates, with the *mia40a* transcript showing the strongest upregulation (3-fold) (Fig 5C).

To further determine the function of differentially expressed genes and proteins we performed a Gene Ontology (GO) enrichment analysis. The upregulated genes were largely involved in amino-acid activation and tRNA metabolic processes (Fig 5D), recapitulating recently reported observations using transcriptome profiling in tissue culture models of MIA dysfunction [52]. Interestingly, proteolysis was identified as the top-rated GO term for downregulated genes (Fig 5D). Enriched proteins were mainly associated with lipid transport and other metabolic processes (Fig 5E). In contrast, downregulated proteins were notably enriched in mitochondrial terms including electron transport chain, cellular respiration and ATP metabolic process (Fig 5E). This observation was supported by steady state level analysis of protein lysates obtained from zebrafish larvae at 5 dpf using antibodies with confirmed specificity in zebrafish (S4 Fig).

### Mia40a loss leads to abnormal expression of liver and pancreas genes

**Fig 6:**
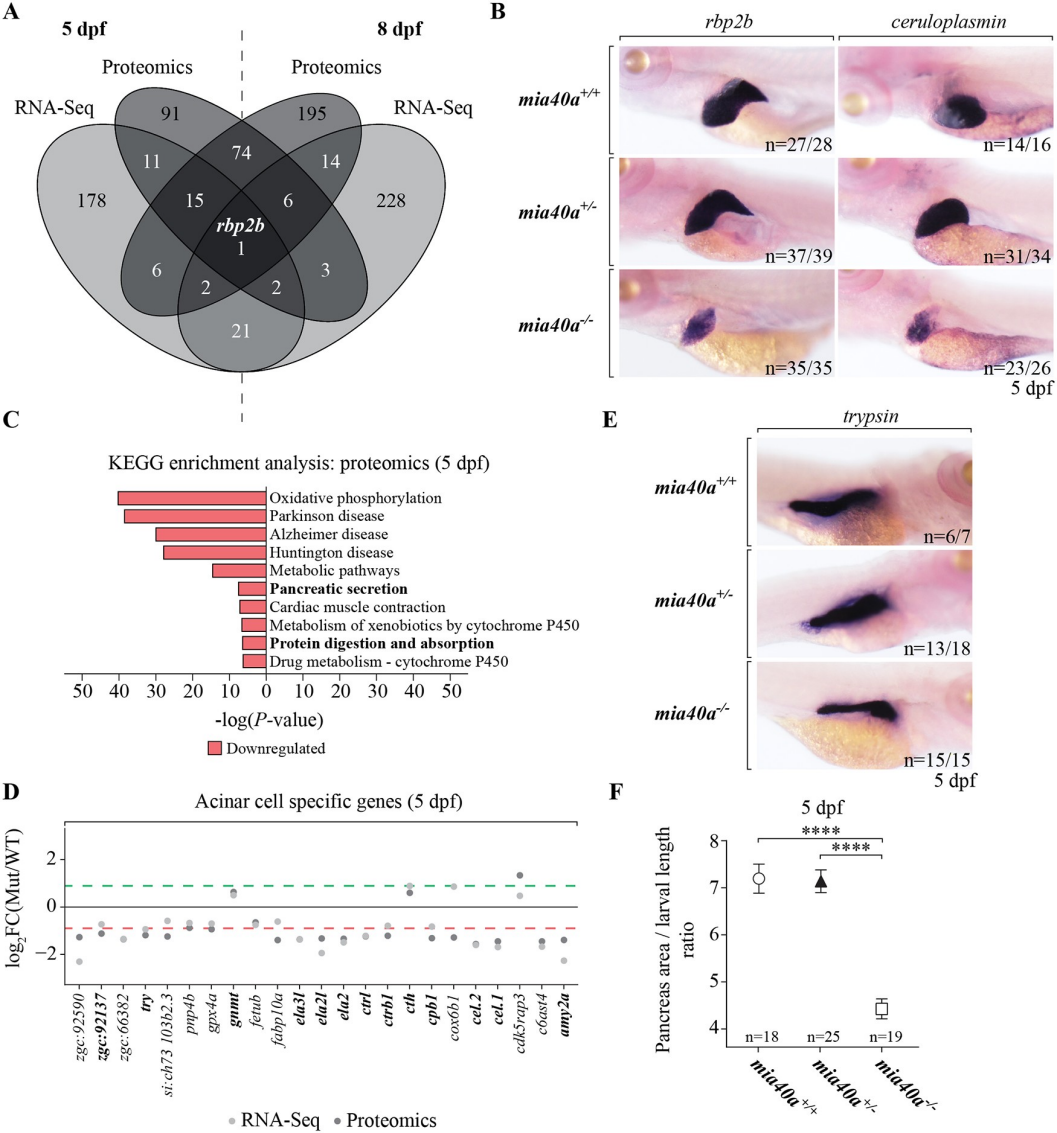
MIA pathway is required for liver and pancreas development. (A) Venn diagram representing differentially expressed genes and proteins (FDR 5%; log_2_FC = < -0.9 and > = 0.9) for 5 and 8 dpf samples. (B) Whole mount *in situ* hybridization demonstrates a decreased expression of *rbp2b* and the liver marker *ceruloplasmin* (*cp*), in the liver of *mia40a* larvae at 5 dpf. n: number of analysed individuals. All images are lateral views, anterior to the left. (C) Enrichment analysis of 155 downregulated proteins in 5 dpf samples using the Kyoto Encyclopedia of Genes and Genomes (KEGG). Results are presented as a negative of log_10_ of *P*-value after Bonferroni correction. Common enriched pathways in RNA sequencing and proteomics are shown in bold. For better representation of the results the bars enriched for downregulated proteins were transformed to be located on the left hand side of the plot. (D) Expression changes of acinar cell specific genes on transcriptomic and proteomic levels. Each value is represented as a log_2_-fold change (log_2_FC) of the expression in mutant samples (Mut) relative to the wild-type (WT) control. The red dashed line corresponds to log_2_FC = -0.9 and the green dashed line to log_2_FC = 0.9. Features highlighted in bold are conserved between zebrafish, mouse and humans [55]. (E) Whole mount *in situ* hybridization with a specific RNA probe shows a decrease in the expression of *trypsin* mRNA in the exocrine pancreas of 5 dpf *mia40a*^*-/-*^ larvae. n: number of analysed individuals. All images are lateral views, anterior to the right. (F) The index of pancreatic area normalized to larva size at 5 dpf is significantly reduced in the homozygous *mia40a* mutants. Error bars correspond to SEM; *P* < 0.0001 (****) by unpaired *t*-test; n: number of analysed individuals.

We next performed an analysis which aimed at detecting tissue and organ-specific phenotypic effects in *mia40a* mutant larvae by comparing differentially expressed features (FDR 5%; log_2_FC = < -0.9 or > = 0.9) for 5 and 8 dpf samples in the transcriptomic and proteomic responses (Fig 6A). The cellular-retinol binding protein 2b (Rbp2b) was identified to be downregulated in all datasets (Fig 6A). Rbp2b plays a key role in retinol homeostasis and is expressed predominantly in the liver [53]. *In situ* hybridization confirmed a substantial decrease in abundance of *rbp2b* mRNA in the *mia40a* mutant liver compared to heterozygous and wild-type siblings (Fig 6B). Following this finding, we observed that the transcript for *ceruloplasmin* (*cp*), a gene encoding a liver-specific marker [54], is also decreased in *mia40a* mutant larvae at 5 dpf (Fig 6B), as were other liver-specific transcripts (S5 Fig). These findings suggest that the changes observed at the global proteomic level could be a consequence of tissue-specific downregulation of the transcript level as exemplified by *rbp2b*.

To further explore biological pathways directly affected by the loss of Mia40a, we performed an enrichment analysis using the Kyoto Encyclopaedia of Genes and Genomes (KEGG). This analysis revealed no enriched pathways for upregulated proteins in 5 dpf samples, whereas interesting results were obtained for depleted proteins (Fig 6C). Oxidative phosphorylation (OXPHOS) was the most affected metabolic pathway, followed by several neurodegenerative diseases that are known to coincide with defects in OXPHOS (Fig 6C). KEGG enrichment analysis also revealed an interesting link between mitochondrial and pancreatic dysfunction. In fact, pancreatic secretion, as well as protein digestion and absorption were also identified among pathways enriched in downregulated genes (in bold Fig 6C, S6 Fig) of 5 dpf samples.

We further explored the resulting hypothesis of pancreatic insufficiency by cross-referencing our results to published transcriptome profiles for pancreatic cell types in zebrafish [55], which mainly include genes encoding various digestive enzymes and their precursors including trypsin (*try*), chymotrypsinogen B1 (*ctrb1*), elastases (*ela2*, *ela2l*, *ela3l*) and pancreatic alpha-amylase (*amy2a*). Interestingly, this analysis yielded 23 acinar-specific genes as downregulated (Fig 6D), 13 (56%) of which are known to be evolutionary conserved across zebrafish, human and mouse (Fig 6D, in bold) [55]. Using a probe against *trypsin* to visualize the exocrine pancreas [56], we validated the omics-derived hypothesis and observed the pancreas to be smaller in *mia40a* mutant larvae as compared to heterozygous and homozygous siblings (Fig 6E). In separate experiments, we measured the pancreas size and normalized it to the larvae body length. We observed a nearly 40% decrease in the pancreas to body length ratio specifically in *mia40a* mutant larvae (Fig 6F), which suggests the difference in pancreatic size does not arise from a general delay in development. In conclusion, we found that Mia40a-induced mitochondrial insufficiency influences the development of endodermal organs such as pancreas.

## Discussion

In this study, we provide evidence for the requirement of a functional MIA pathway for zebrafish development and survival. We have shown that both paralogues of zebrafish *mia40* appear to retain their activity as they sustain growth of yeast strains which lack endogenous *MIA40* (Fig 1B) [14], providing evidence for MIA conservation during evolution. We found that expression of *mia40a* suffice for zebrafish development and survival in conditions when *mia40b* is mutated, but not *vice versa*, suggesting a non-redundant role for the paralogues. We think that the lethal phenotype of *mia40a* mutants can be explained by the fact that the *mia40a* transcript is expressed more abundantly and in more tissue types in zebrafish (Fig 1C–1E). Nonetheless, by overexpressing wild-type *mia40b* we were able to rescue the appearance of GFP-positive inclusions in the *mia40a* mutants in the transgenic background (S2B Fig).

Mia40 deletion in mice causes respiratory chain complex CI-20 defects and developmental arrest at gastrulation [20], whereas our zebrafish *mia40a* mutant does not show any strong retardation until much later in development (Fig 2B and 2C). The phenotypic differences between mice and zebrafish, observed also in mtDNA polymerase gamma (*polg*) mutants [33, 57], are interesting and difficult to explain. One can hypothesize that zebrafish embryos, through the adaptation to the water environment, can better tolerate low oxygen levels [58, 59] and as such do not rely on cellular respiration during early development to the extent that the mice do. Additionally, the zebrafish zygote is preloaded with maternally-contributed mitochondria and *mia40a* transcript (Fig 1C). When combined, these effects possibly suffice for the initial steps of embryogenesis. This view is supported by the observation that mtDNA copy number drastically decreases during the first 24 hpf and genes responsible for mtDNA replication are expressed later in development [60]. Another possibility is the ability of the developing zebrafish embryo to retrieve energy from other sources, including glycolysis. In support of this hypothesis, we observe that *mia40a* mutants respire at levels comparable to the heterozygous and wild-type siblings at 5 dpf (Fig 4A), yet the mutants have to take advantage of the entire respiratory capacity of mitochondria to achieve that (Fig 4A) and already utilize glucose as an energy source (Fig 4B and 4C). Surprisingly, we observed an increase in the mtDNA content at this time point (S3A Fig). It is intriguing to hypothesize that by upregulating mtDNA synthesis, *mia40a* mutants attempt to compensate for reduced mitochondrial respiration capacity, compromised by insufficient import of MIA substrates. Whether the enlarged mitochondria structures in the skeletal muscle of the zebrafish mutant represent a further compensatory mechanism is an interesting possibility that may gain support from previous studies, which showed that mitochondrial fusion has a protective function in sustaining mtDNA stability in the skeletal muscle [61].

Our transcriptomics and proteomics data point to a stronger mitochondrial-specific response at the protein level (Fig 5B), in line with a recent omics report of hearts from mouse mitochondrial mutants [62] and studies which modelled compromised mitochondria using various stressors in mammalian cell culture [52]. We provide an elegant validation of the cellular respiration phenotype by our proteomics data enrichment analysis (Fig 5E) and provide a more specific analysis of Mia40 substrates, which are strongly downregulated at the protein level in our zebrafish mutant (Fig 5C). Interestingly, the transcripts presented the opposite tendency, with mutated *mia40a* transcript being most significantly increased (Fig 5C). This may suggest yet another level of attempted compensation for the loss of Mia40a activity.

We were surprised to not observe acute molecular hallmarks of programmed cell death in the 8 dpf enrichment analysis in the *mia40a* mutants, suggesting that the previously reported interaction between Mia40 and AIF plays only a role in mitochondrial bioenergetics [20]. Lack of apoptotic signalling may also be explained by the fact that whole larvae were analysed, which hampers the detection of processes in specific or more strongly affected tissues. Based on metabolite analysis (S3B and S3C Fig), we expected the 8 dpf larvae to undergo starvation and thus focused our interpretation on the 5 dpf data. From our omics approaches it is evident that endodermal organs such as the liver and pancreas are affected in the *mia40a*^*-/-*^ larvae already at that stage. It has previously been reported that mutations in the gene *tomm20*, which encodes a component of the protein translocase of the outer mitochondrial membrane, plays a role in hepatocyte survival in zebrafish [30]. Moreover, studies that aimed at transient knock-down of *Erv1/ALR*, the partner of Mia40, reported a role in liver and pancreas development in zebrafish [36]. Here, we expand on these reports and show that the acinar cells specifically are the cell type most strongly affected in the *mia40a* mutant (Fig 6D).

The question remains why acinar cells specifically suffer most prominently from MIA dysfunction. Mitochondria are grouped in three functionally distinct subpopulations in these highly polarized cells, forming perigranular, perinuclear and subplasmalemmal mitochondria [63, 64]. This mitochondrial compartmentalization is of physiological importance and enhances optimal Ca^2+^ signalling and ATP supply, which are instrumental in governing the basic function of the exocrine gland [63, 65]. We thus hypothesize that MIA dysfunction can contribute on both the functional and structural level to the acinar cell-specific phenotype in our mutant. First, we have shown that mitochondrial function is compromised in our mutant (Fig 4 and S3 Fig) and we expect that cells and tissues that strongly rely on mitochondrial biogenesis to be most affected by functional shortage. In support, it has been reported that genes responsible for mtDNA replication are expressed in the exocrine pancreas from 4 dpf to meet the increased demand for mitochondrial biogenesis in this organ at this developmental stage [60]. The authors suggest an interesting idea that this boost in expression of mtDNA replication genes can enhance mitochondrial biogenesis to support the excessive adaptation of the exocrine organ for the hydrolysis of external food [60]. Second, Mia40 has been shown to control the biogenesis of MICU1, a regulator of the mitochondrial Ca^2+^ uniporter (MCU) responsible for mitochondrial calcium intake [66]. Thus, the *mia40a* mutation could trigger functional abnormalities especially in Ca^2+^ signalling-dependent acinar cells. Finally, several mutants identified in ENU mutagenesis screens showed a smaller pancreas phenotype [67–69]. Interestingly, the *mitomess* (*mms*) zebrafish mutant, which presents dileted mitochondria in acinar cells, was additionally characterised with decreased levels of acinar-specific markers, as is observed in our *mia40a*^*-/-*^ model [67]. The gene underlying the *mitomess* phenotype has not yet been identified and it is intriguing to speculate it may be a component of the MIA pathway [67].

Dysfunction of acinar cells and the exocrine pancreas as observed in this study have been previously reported as resulting from mutations in mitochondrial-encoded proteins, including in such pathologies as Pearson’s syndrome [70–72]. The model of mitochondrial insufficiency presented here shares additional symptoms with those syndromes including lactic acid accumulation and nutrient malabsorption [70, 72]. To our knowledge, this is the first time these clinical features are linked to a mutation in a nuclear-encoded mitochondrial gene. Our data may thus contribute to a better understanding of the organismal consequences of mitochondrial pathologies, and enhance a more accurate diagnosis and advancement of treatment strategies in the future.

## Materials and methods

### Nomenclature

There are two paralogues of the *mia40* (*chchd4*) gene in the zebrafish genome, *mia40a* (*chchd4a*, ENSDARG00000033376) and *mia40b* (*chchd4b*, ENSDARG00000040707).

### Zebrafish handling and ethics statement

Zebrafish husbandry was performed in accordance with institutional and national ethical and animal welfare guidelines. Procedures involving animals were approved by the First Warsaw Local Ethics Committee for Animal Experimentation (357/2012 and 197/2016), the Veterinary Department of the Regional Council of Darmstadt (B2/1017) and the Medical University of South Carolina Institutional Animal Care and Use Committee (AR #2850).

Mutant lines were generated in the AB background. The *Tg(Xla*.*Eef1a1*:*mlsEGFP)*^*cms1*^ zebrafish line was used in this study [47].

### Generation and identification of zebrafish mutants

#### Targeted mutagenesis using TALENs

TALEN targeter software (https://tale-nt.cac.cornell.edu) was used to design and guide endonucleases to the genomic loci of interest. TALENs were constructed using the Golden Gate assembly [73, 74]. TALEN arm-recognised DNA sequences marked on Fig 2A (*mia40a*) and Fig 2B (*mia40b*). A maximum of 400 pg of total TALEN RNA was injected into the cell of a 1-cell stage zebrafish embryo.

#### gDNA isolation and genotyping

To test TALEN efficiency, genomic DNA (gDNA) was extracted from individual TALEN-injected embryos. In brief, 24 hpf embryos were suspended in 27 μl of 50 mM NaOH and incubated at 95°C for 10 min followed by incubation on ice. Next, 3 μl of 1 M Tris-HCl, pH 8.0 was added to reach the final concentration of 0.1 M Tris-HCl, pH 8.0 [75]. The gDNA in conjunction with the DyNAmo SYBR green (Thermo Fisher Scientific) and primers flanking the targeted loci (S3 Table) were used for PCR reactions and high-resolution melt analysis (HRMA) using the Eco Real-Time PCR System (Illumina) [76]. The PCR reaction conditions were as follows: 95°C for 15 s, then 40 cycles of 95°C for 10 s, 60°C for 15 s. The HRMA curve was generated by collecting SYBR-green fluorescence data in the 65–95°C range. Upon confirmation of functional TALENs, the F0 siblings were raised to adulthood and outcrossed to identify founders. The F1 generation was raised to adulthood and subjected to caudal fin-clip to obtain gDNA for heterozygous fish identification by HRMA. The mutation carrying PCR products were cloned into pGEM-T Easy (Promega) for sequencing.

#### Genotyping by fin-fold amputation

Where indicated, larvae were genotyped at 72 hpf by fin-fold amputation. Briefly, upon anaesthesia in 0.04% Tricaine solution, fin-folds were amputated from individual larvae under the stereomicroscope (Stemi 2000, Zeiss). Tissue was collected in 6.3 μl of 50 mM NaOH and incubated at 95°C for 10 min followed by incubation on ice. pH was neutralized by adding 0.7 μl of 1 M Tris-HCl pH 8.0 to reach the final concentration of 0.1 M Tris-HCl, pH 8.0 [75]. 2 μl of the isolated gDNA was used in the PCR and HRMA reaction with specific primers (S3 Table).

### Yeast complementation assay

#### Generation of constructs and yeast strains expressing *Danio rerio mia40* homologues

Coding sequences (CDS) from *Danio rerio mia40a* and *mia40b* were amplified by PCR from cDNA (primers are listed in S3 Table). *mia40a* or *mia40b* were inserted into pFL39 vector by restriction digest using KpnI and XhoI restriction sites. The resulting constructs express *Danio rerio mia40a* (#349) or *mia40b* (#348) under the endogenous yeast *MIA40* promoter and terminator. To express *Danio rerio’s* Mia40a protein version with mitochondrial targeting b2 bipartite signal sequence (1-84aa), *mia40a* cDNA was cloned into pFL39 plasmid already containing *b2* presequence by restriction digest using the restriction sites BamH1 and XhoI. The resulting construct expresses *b2*_ *mia40a* (#352) under the endogenous yeast *MIA40* promoter and terminator. To express Mia40b protein with mitochondrial targeting sequence, *b2* presequence was amplified (1-84aa) and fused by PCR to the cDNA sequence of *Danio rerio mia40b*. The fusion product was inserted into pFL39 plasmid by restriction digest using KpnI and XhoI. The resulting construct expresses *b2*_*mia40b* under the control of endogenous yeast *MIA40* promoter and terminator (#353).

The *S*. *cerevisiae* strains used are derivatives of YPH499 (MATa, ade2-101, his3-Δ200, leu2-Δ1, ura3-52, trp1-Δ63, lys2-801). The pFL39 derived plasmids were transformed into yeast with chromosomal deletion of yeast *MIA40* expressing *MIA40* from plasmid (#182). Genetic manipulations and plasmid shuffling were performed according to standard procedures. Resulting yeast strains containing pFL39 derived plasmids with *mia40a* (#1084), *mia40b* (#1086), *b2* (1–84)_*mia40a* (#1085) and *b2* (1–84)_ *mia40b* (#1087). The yeast strain containing wild-type *MIA40* was described previously (#398) [14].

#### Yeast growth conditions

For drop tests, yeast were grown in selective minimal medium (0.67% (w/v) yeast nitrogen base, 0.074% (w/v) CSM-TRP, 2% glucose) and serial dilutions were spotted on minimal medium agar plates (0.67% yeast nitrogen base supplemented with appropriate amino acids and nucleotides, 2% glucose) with or without 5-fluoroorotic acid (FOA) and grown at 24°C.

### RNA isolation and RT-qPCR

#### Total RNA isolation

Total RNA was isolated from 5 larvae and pooled according to genotype at indicated time points. Briefly, larvae were suspended in 200 μl of Trizol (Ambion) and frozen at -80°C for at least 24 h. Upon defrosting, sterile zirconium oxide beads were added to the samples and tissue was disrupted using the Bullet Blender 24 Gold (Next Advance) for 5 min at speed level 8. Samples were spun briefly and the supernatant was subjected to phenol-chloroform extraction. DNA contaminants were removed by DNase treatment (Promega). RNA was concentrated using RNA Clean and Concentration kit (Zymo Research). RNA quality and quantity were assessed using the Nanodrop 2000c spectrophotometer (Thermo Fisher Scientific).

#### RT-qPCR

500 ng of obtained RNA was reverse transcribed to cDNA using High-Capacity RNA-to-cDNA kit (Applied Biosciences). All procedures were carried out in a clean environment with nuclease-free water (Invitrogen) and pipette tips. At least two primer pairs were designed for the target of interest. Melting temperature and specificity of primers was tested, and only one pair of primers was selected for subsequent analysis (S3 Table). Serial dilution of cDNA was performed in conjunction with specific primers to obtain dynamic range conditions. DyNAmo SYBR green reagent and 0.5 μM final primers concentration was used in qPCR reactions, performed in a Bio-Rad CFX Connect real-time instrument with following conditions: 95°C for 7 min, then 40 cycles of 95°C for 10 s, 60°C for 15 s. The HRMA curve was generated by collecting SYBR-green fluorescence data in the 60–92°C range. mRNA levels for *mia40a* and *mia40b* were relatively quantified to the level of *rpl13a*. A variation of Livak method was used and the values were presented as 2^^(-ΔC^_T_^)^ using *rpl13a* as a reference gene [77].

### *In situ* hybridization

Whole-mount *in situ* hybridization was performed essentially as described [78] with the following changes: 5 dpf larvae were permeabilized in 40 μg/ml proteinase K for 20 min, and probes were used at a concentration of 1 μg/ml in hybridization solution. Images were acquired using a Nikon SMZ25. Contrast and brightness were adjusted using Adobe Photoshop. *In situ* primers are listed in S3 Table.

### RNA rescue experiments

Full-length wild-type and mutated CDS of *mia40a* a *mia40b* were amplified from *mia40a*^*+/bns292*^ and *mia40b*^*+/bns293*^ cDNA using specific primers (S3 Table). Amplicons were cloned into pCS2+ plasmids linearized with BamH1 using Cold Fusion (System Biosciences) and sequenced to confirm correct inserts. Plasmids were linearized with Not1 and mRNA was transcribed using the mMESSAGE mMACHINE SP6 synthesis kit (Ambion), followed by TURBO DNase treatment. mRNA was purified using the RNA Clean-Up and Concentrator kit (Zymo Research) and mRNA quality was assessed using the Nanodrop 2000c spectrophotometer (Thermo Fisher Scientific) and agarose gel electrophoresis. Different amount of wild-type versions, ranging from 50 to 500ng, were injected at 1-cell stage into embryos from a heterozygous in-cross of *mia40a*^*+/bns292*^ larvae in the *Tg(Xla*.*Eef1a1*:*mlsEGFP)* transgenic background. The highest dose of mRNA that did not trigger a severe phenotype in larvae was chosen for the experiments. At 3 dpf, larvae were embedded in 1% low-melting agarose containing 0.04% Tricaine solution in a glass-bottom dish (MatTek Corporation, Ashland, MA, USA) and analysed using the LSM800 confocal laser scanning microscope (Zeiss).

### Imaging

#### Phalloidin staining

To visualise actin fibres, 1-phenyl-2-thiourea (PTU)-treated larvae were fixed with 4% paraformaldehyde (PFA, Sigma) overnight (O/N) at 4°C. Next, samples were washed thrice with phosphate-buffered saline (PBS, Sigma) containing 0.2% Triton X-100 (0.2% PBSTx) for 5 min each at room temperature (RT), permeabilized in 2% PBSTx for 2 hours at RT and again washed trice with 0.2% PBSTx for 5 min each at RT. Samples were incubated in 0.2% PBSTx containing 1:100 dilution of Alexa 568-coupled Phalloidin (Molecular Probes), O/N at 4°C. The next day, samples were washed five times with 0.2% PBSTx for 5 min each. Larvae were stored in 0.2% PBSTx at 4°C until imaging. All incubation steps and washes were done on the rocker and samples were protected from light. Larvae were embedded in 1% low-melting agarose in a glass-bottom dish (MatTek Corporation, Ashland, MA, USA). Images were acquired using the LSM880 confocal laser scanning microscope (Zeiss).

#### Tomm20 staining

For immunostaining with the anti-Tomm20 antibody (FL-145, Santa Cruz Biotechnology) larvae were fixed with 4% PFA O/N at 4°C. The next day samples were washed thrice with Dulbecco’s phosphate-buffered saline (D-PBS, Sigma) containing 0.5% Triton X-100 (0.5% D-PBSTx) for 5 min at RT and skinned manually. Samples were fixed in 4% PFA for 20 min at RT followed by washing thrice with 0.5% D-PBSTx for 5 min at RT. Blocking was done in buffer containing 1% DMSO (Sigma) and 10% Goat serum (Gibco) in 0.5% D-PBSTx for 1 h at RT. The primary antibody was diluted 1:150 in the blocking buffer and samples were incubated O/N at 4°C. Samples were washed thrice with 0.5% D-PBSTx for 5 min at RT and blocked by incubation in blocking buffer for 1 h at RT. Samples were incubated with Alexa 568-coupled goat anti-rabbit secondary antibody (Molecular Probes) diluted 1:500 in blocking buffer, O/N at 4°C. DNA was counterstained with DAPI (Sigma) diluted 1/5000 in 0.5% D-PBSTx, for 15 min at RT. Samples were washed thrice with 0.5% D-PBSTx for 1 h each. Larvae were stored in D-PBS at 4°C, until imaging. All incubation steps and washes were done on the rocker and samples were protected from light. Larvae were embedded in 1% low-melting agarose in a glass-bottom dish (MatTek Corporation, Ashland, MA, USA). Images were acquired using the LSM800 confocal laser scanning microscope (Zeiss).

#### Transmission electron microscopy (TEM)

5 dpf wild-type and *mia40a* mutant larvae were fixed in 2% v/v glutaraldehyde in 0.05 M sodium phosphate buffer (pH 7.4) for 24 h. Samples were rinsed three times in 0.15 M sodium cacodylate buffer (pH 7.4) and subsequently post fixed in 1% (w/v) OsO_4_ and 0.05 M potassium ferricyanide in 0.12 M sodium cacodylate buffer (pH 7.4) for 2 h. The specimens were dehydrated in a graded series of ethanol, transferred to propylene oxide and embedded in Epon according to standard procedures [79]. Sections approximately 80 nm thick were cut with a Leica UC7 microtome and collected on copper grids with Formvar supporting membranes. Sections were stained with uranyl acetate and lead citrate and subsequently examined with a Philips CM 100 transmission emission microscope (Philips, Eindhoven, The Netherlands), operated at an accelerating voltage of 80 kV. Digital images were recorded with an OSIS Veleta digital slow scan 2k x 2k CCD camera and the ITEM software package. Images were analysed using ImageJ, Fiji.

### Larval respiration assay

Oxygen consumption rate (OCR) measurements from zebrafish at 5 and 10 dpf were performed using the XF-24 Extracellular Flux Analyzer (Seahorse Bioscience) with slight modifications of the standard method [80]. Briefly, fish were placed individually in 20 wells of a 24-well islet plate (Seahorse Bioscience) and kept in place with an islet capture screen. Basal respiration was calculated for each animal from the two consecutive OCR readings prior to injection of 1.5 μM carbonyl cyanide-4-(trifluoromethoxy)phenylhydrazone (FCCP). Maximal FCCP-uncoupled respiration was determined by averaging the maximum OCR after injection for each larva. Each individual was harvested after respirometry and genotyped as above. Nonparametric comparisons for each genotype pair were conducted for basal respiration, maximal respiration, and spare respiratory capacity (SRC, the result of subtracting basal respiration from maximal respiration), with the Wilcoxon method using JMP software.

### Metabolite measurements

Measurements of total body glucose and lactate were performed on a pool of 5 larvae per genetic condition using a fluorescence-based enzymatic detection kit (Biovision Inc.) as described before [81]. Briefly, larvae were collected in 1.5 ml microcentrifuge tubes, the excess of water was removed and samples were frozen stored at -80°C until further analysis. Upon defrosting, 15 μl PBS per larvae was added and tissues were disrupted with a sterile pestle (Axygen), on ice. Samples were spun at 14,000 x g for 10 min, at 4°C. The supernatant was immediately used for metabolite measurements according to manufacturers’ protocol. Fluorescence readings (Ex/Em 535/590) were taken with the FLUOstar Omega instrument (BMG Labtech). Results were read from a linear regression curve based on the dilutions of the standard. Statistical analysis between indicated groups was performed using the unpaired *t*-test and considered significant at *P* < 0.05 (*), *P* < 0.01 (**) and *P* < 0.001 (***).

### mtDNA levels

Zebrafish larvae were genotyped by fin-fold amputation at 3 dpf. Whole DNA extraction was performed from individual larva of indicated genotype using the DNeasy Blood and Tissue kit according to manufacturers’ protocol (Qiagen). The concentration and quality of isolated DNA was assessed using the Nanodrop 2000c spectrophotometer (Thermo Fisher Scientific). 5 ng of obtained DNA was used in qPCR reactions according to previously described protocol (refer to RT-qPCR subchapter). Primers specific to mitochondrial-encoded NADH-ubiquinone oxidoreductase chain 1 (*mt-nd1*) and nuclear-encoded DNA polymerase gamma (*polg*) for control were used (S3 Table). Statistical analysis between indicated groups was performed using the unpaired *t*-test and considered significant at *P* < 0.05 (*) and *P* < 0.01 (**).

### SDS-PAGE and western blotting

Protein was extracted from pools of at least 20 larvae at 5 dpf. Larvae were lysed in buffer containing 20 mM Tris-HCl pH 7.4, 200 mM NaCl, 0.5% Triton X-100 and 2 mM PMSF using a sterile pestle to disrupt the tissue (Axygen). The samples were centrifuged at 3000 x g for 5 min at 4°C and lysates were mixed with same volume of chloroform/methanol (2:1) solution. Upon vortexing, one volume of 10% TCA diluted in cold 100% acetone was added and samples were precipitated on ice for 5 min. Upon centrifugation at 10,000 x g at 4°C for 1 min the upper, aqueous phase was discarded and 2 volumes of 10% TCA in cold dH_2_O was added to the samples and vortexed. The 10% TCA wash was repeated thrice. Next, 5 volumes of cold dH_2_O was added followed by centrifugation at 10,000 x g at 4°C for 1 min and addition of 1 ml of ice cold 100% acetone. Upon another centrifugation step, samples were washed with 80% acetone. Pellets were dissolved in 75 μl of sample buffer containing 30 mM Tris-HCl pH 6.5 and 4% SDS and heated to 70°C. For equal loading, protein concentration was established using Direct Detect spectrometer (Millipore). Samples were mixed with Laemmli buffer containing 50 mM DTT and incubated for 15 min at 65°C. 50 μg protein sample was subjected to SDS-PAGE and western blotting in conjunction with specific antibodies: anti-Ndufa9 (ab14713, 1:300 dilution), anti-Cox4i1 (ab14744, 1:1000 dilution), anti-Atp5a (ab14748, 1:4000 dilution), anti-Rpl7 (A300-741A, 1:000 dilution), anti-β Actin (A1978 Sigma Aldrich, 1:1000 dilution).

### RNA-Seq analysis

For RNA-Seq, RNA was isolated from a pool of 5 larvae at 5 and 8 dpf using the miRNeasy micro Kit (Qiagen) combined with on-column DNase digestion (DNase-Free DNase Set, Qiagen) to avoid contamination by genomic DNA. RNA and library preparation integrity were verified with a BioAnalyzer 2100 (Agilent) or LabChip Gx Touch 24 (Perkin Elmer). 1–2 μg of total RNA was used as input for Truseq Stranded mRNA Library preparation following the low sample protocol (Illumina). Sequencing was performed on the NextSeq500 instrument (Illumina) using v2 chemistry, resulting in average of 20 M reads per library with 2 x 75 bp paired end setup. The resulting raw reads were assessed for quality, adapter content and duplication rates with FastQC [82]. Trimmomatic version 0.33 was employed to trim reads after a quality drop below a mean of Q18 in a window of 5 nucleotides [83]. Only reads above 30 nucleotides were cleared for further analyses. Trimmed and filtered reads were aligned versus the Ensembl Zebrafish genome version DanRer10 (GRCz10.87) using STAR 2.4.0a with the parameter “—outFilterMismatchNoverLmax 0.1” to increase the maximum ratio of mismatches to mapped length to 10% [84]. The number of reads aligning to genes was counted with featureCounts 1.4.5-p1 tool from the Subread package [85]. Only reads mapping at least partially inside exons were admitted and aggregated per gene. Reads overlapping multiple genes or aligning to multiple regions were excluded. 8 dpf samples were collected and processed on different time-points and a significant batch effect was noticed for these samples. No such effect was noticed for 5 dpf samples, collected and processed simultaneously. The batch effect correction for 8 dpf samples was done using removeBatchEffect function from edgeR package version 3.16.5 on log-transformed expression matrix (S4 Table) with a minimum combined mean of 5 reads. The result of batch removal is presented on the S7 Fig and the expression matrix with no batch effect is available in S5 Table. Pearson correlation analysis revealed high reproducibility of biological replicates with correlation coefficient values > 97% for transcriptomic data (S8 Fig). Differentially expressed genes were identified using DESeq2 version 1.62 [86]. Only genes with a minimum fold change of log_2_ = < -0.9 and > = 0.9, a maximum Benjamini-Hochberg corrected *P*-value of 0.05, and a minimum combined mean of 5 reads were deemed to be significantly differentially expressed (S1 Table). The Ensemble annotation was enriched with UniProt data (release 06.06.2014) based on Ensembl gene identifiers (IDs) [87]. Raw data were deposited to GEO repository with the accession number GSE113272.

### Mass spectrometric analysis

#### Sample preparation

Protein was extracted from pools of 5 larvae at 5 and 8 dpf. Larvae were lysed in SDS buffer (4% SDS in 0.1 M Tris/HCl, pH 7.6) using a sterile pestle to disrupt the tissue (Axygen). Samples were heated to 70°C for 10 min. DNA was sheared by sonication and cell debris was removed by centrifugation at 14.000 x g for 10 min. The colorimetric 660 nm protein assay (Pierce) was used to determine the concentration of solubilized proteins in the supernatants. Proteins were subsequently precipitated by addition of four sample volumes of ice-cold acetone and incubation at -20°C overnight, followed by pelleting at 14.000 x g for 10 min and washing of the pellet with 90% acetone. Samples were dried to remove residual acetone and dissolved in urea buffer (6 M urea, 2 M thiourea, 10 mM HEPES, pH 8.0). In-solution digest was performed to enzymatically digest the proteins [88]. First, protein disulfide bonds were reduced using 4 mM dithiothreitol (DTT) and cysteines subsequently alkylated using 20 mM iodoacetamide (IA). Next, proteins were cleaved using Lys-C (50:1 protein-to-enzyme ratio; Wako Chemicals GmbH) at room temperature for 3 hours, followed by overnight trypsination (50:1 protein-to-enzyme ratio; Serva) at room temperature. The Fluorimetric Peptide Assay (Pierce) was used to estimate peptide concentration in trypsin-digested samples. Samples containing equal amounts of peptides (65 μg) were subjected to a dimethyl (in-solution) labeling protocol as previously described [89]. In brief, the N-termini and lysine residues were methylated for 1 h in RT by formaldehyde-H_2_ and cyanoborohydride (light, *mia40a*^*+/+*^) and formaldehyde-^13^C–D_2_ and cyanoborodeuteride (heavy, *mia40a*^*-/-*^), respectively (all reagents: Sigma). Upon quenching the reaction and acidification, differentially labeled samples were mixed at a 1:1 ratio. Resulting samples were fractionated using the high pH reversed-phase peptide fractionation kit (Pierce) according to the manufacturers’ protocol.

### Liquid chromatography/tandem mass spectrometry (LC/MS^2^)

Fractionated peptides were reconstituted in 10 μl of solvent A (0.1% formic acid) and subjected to mass spectrometric analysis in line to reversed phase capillary chromatography. Peptides were separated using an UHPLC system (EASY-nLC 1000, ThermoFisher Scientific) and 20 cm in-house packed C18 silica columns (1.9 μm C18 beads, Dr. Maisch GmbH) coupled to a QExactive HF orbitrap mass spectrometer (ThermoFisher Scientific) using an electrospray ionization source. The gradient employed consisted of linearly increasing concentrations of solvent B (90% acetonitrile, 1% formic acid) over solvent A (5% acetonitrile, 1% formic acid) from 5% to 30% over 215 min, from 30% to 60%, from 60% to 95% and from 95% to 5% for 5 min each, followed by re-equilibration with 5% of solvent B. The constant flow rate was set to 400 nl/min.

Full MS spectra were collected for a mass range of 300 to 1750 m/z with a resolution of 60,000 at 200 m/z. The ion injection target was set to 3 x 10^6^ and the maximum injection time limited to 20 ms. Ions were fragmented by higher energy collision dissociation (HCD) using a normalized collision energy of 27, an isolation window width of 2.2 m/z and an ion injection target of 5 x 10^5^ with a maximum injection time of 20 ms. Precursors characterised with unassigned charge state and a charge state of 1 were excluded from selection for fragmentation. The duration of dynamic exclusion was 20 s. Resulting tandem mass spectra (MS/MS) were acquired with a resolution of 15,000 at 200 m/z using data dependent mode with a top 15 loop count.

### Data analysis

MS raw data were processed by MaxQuant (v. 1.6.0.1) [90] using the Uniprot zebrafish database (as of 17.08.2017) containing 59064 entries and the following parameters: a maximum of two missed cleavages, mass tolerance of 4.5 ppm for the main search, trypsin as the digesting enzyme, carbamidomethylation of cysteines as a fixed modification and oxidation of methionine as well as acetylation of the protein N-terminus as variable modifications. For the dimethyl-labeled protein quantification, isotope labels were configured for peptide N-termini and lysine residues with a monoisotopic mass increase of 28.0313 and 36.0757 Da for the light and heavy labels, respectively. Peptides with a minimum of seven amino acids and at least one unique peptide were included in the analysis. MaxQuant was set to filter for 1% false discovery rate at the peptide and protein levels, both. Only proteins with at least two peptides and one unique peptide were considered identified and included in further data analysis (S6 Table). The mass spectrometry proteomics data have been deposited to the ProteomeXchange Consortium via the PRIDE partner repository with the dataset identifier PXD009594 [91, 92].

The obtained data was subjected to differential expression analysis using the in-house R package autonomics (https://github.com/bhagwataditya/autonomics; version 1.0.21), which makes use of functionality provided by limma (S1 Table) [93].

### KEGG and GO enrichment analysis

Gene Ontology (GO) and Kyoto Encyclopaedia of Genes and Genomes (KEGG) enrichment analysis was performed for RNA sequencing and quantitative proteomics data using KEGG.db (v. 3.2.3) and GO.db (v. 3.4.1) R/Bioconductor packages. The KEGG enrichment analysis was done using human orthologue genes from package org.Hs.eg.db (v 3.4.0), while GO enrichment analysis using zebrafish data from package org.Dr.eg.db (v 3.4.1). Pathways and genes selected in each developmental stage were filtered after Benjamini-Hochberg correction for an adjusted *P*-value < 0.05.

### Integration of transcriptomics and proteomics data

The list of expressed proteins (S6 Table) was combined with transcriptomic data. The first gene name in a protein group was used to merge the proteomic data with Ensembl gene IDs for zebrafish. Next, this set was combined with the transcriptomic data by merging on Ensembl gene IDs (S7 Table). The identification of genes encoding mitochondrial proteins using the MitoCarta 2.0 gene set [94] was based on zebrafish Ensembl gene IDs as well (S2 Table).

### Measuring the pancreas area to larvae length ratio

PTU-treated larvae were fixed with 4% paraformaldehyde (PFA, Sigma) overnight (O/N) at 4°C. Next, samples were washed thrice with phosphate-buffered saline (PBS, Sigma). Larvae were embedded laterally, in 1% low-melting agarose in a glass-bottom dish (MatTek Corporation, Ashland, MA, USA). Images were acquired using a Nikon SMZ25. Measurements were done blindly and manually using the NIS Elements software. To obtain the ratio, the pancreas area [μm^2^] was divided by larvae length [μm].

## Supporting information

S1 Fig Mia40 is conserved between species. (A) Protein alignment of zebrafish Mia40a and Mia40b with human, mouse, frog and yeast orthologs. Compared to higher eukaryotes, the yeast ortholog contains a large N-terminal presequence and transmembrane domain. The conserved CPC motif is shown in red and the cysteines arranged in the double CX_9_C motifs in green. The conserved phenylalanine, important for substrate binding, is presented in blue. (B) Phylogenetic analysis of zebrafish Mia40a and Mia40b.
(TIF)

S2 Fig Injections of wild-type but not mutant *mia40a* or *mia40b* mRNA can rescue the appearance of GFP-positive inclusions in the skeletal muscle of *mia40a* mutant larvae. 125 pg of *mia40a*^*+*^ o*r mia40a*^*bns292*^ mRNA (A) or 250 pg of *mia40b*^*+*^ or *mia40a*^*bns293*^ *m*RNA (B) was injected at 1-cell stage into embryos from a heterozygous in-cross of *mia40a*^*+/-*^ in the transgenic background *Tg(Xla*.*Eef1a1*:*mlsEGFP*). The appearance of GFP-positive inclusions in the skeletal muscle of the *mia40a*^*-/-*^ larvae at 3 dpf is rescued by injections of the wild-type (A, B, left panels) but not mutant (A, B, right panels) versions of either protein. To better visualize the mitochondrial morphology, a magnified image is presented on the side. Single plane sections are shown. The arrowhead points to a GFP-positive inclusion. Scale bar, 50 μm; n: number of analysed individuals. All images are lateral views, anterior to the left.
(TIF)

S3 Fig Mutations in *mia40a* trigger respiration defects and a metabolic shift. (A) mtDNA copy number was assayed and expressed as the level of the mitochondria-encoded *mt-nd1* gene normalized to the nuclear-encoded *polg*. Larvae with homozygous mutations in the *mia40a* gene have elevated levels of mtDNA. Data derived from three biological replicates. Error bars correspond to SEM, *P* < 0.05 (*) and *P* < 0.01(**) by unpaired *t*-test. (B-C) Embryos obtained from an in-cross of heterozygous *mia40a*^*+/-*^ siblings were continuously fed from 5 dpf or starved. At 10 dpf, the larvae were collected and subjected to respiration analysis using the Seahorse technology (B) or glucose analysis (C). (B) No significant changes in cellular respiration between the compared genotypes are noticed at 10 dpf upon starvation. Nutrient supply results in elevated respiration in the wild-type and heterozygous siblings, but not in *mia40a* homozygous mutants. Error bars correspond to SEM, *P* < 0.05 (*) by Mann-Whitney test. (C) Preceding death at 10 dpf, glucose levels in *mia40a* homozygous mutants are barely detectable. Data derived from three biological replicates. Error bars correspond to SEM, *P* < 0.01 (**) by unpaired *t*-test.
(TIF)

S4 Fig The steady-state levels of respiratory complex components are decreased in *mia40a* mutants. Protein lysates from wild-type or *mia40a* mutants were subjected to SDS-PAGE analysis and western blotting using specific antibodies against mitochondrial and cytosolic proteins. Representative images for three biological replicates show decreased levels of members of respiratory complex 1 (Ndufa9) and complex 4 (Cox4i1).
(TIF)

S5 Fig Transcriptomic analysis of liver-expressed genes. Expression levels of liver specific genes in 5 dpf samples (FDR 5%). The red dashed line represents log_2_FC = -0.9 and the green dashed line represents log_2_FC = 0.9.
(TIF)

S6 Fig KEGG enrichment analysis for downregulated genes in 5 dpf samples. The results are presented as a negative of log_10_ of *P*-value after Bonferroni correction.
(TIF)

S7 Fig Batch effect removal for 8 dpf RNA-Seq set of samples. (A) Hierarchical clustering of 8 dpf RNA-Seq samples with batch effect. (B) Hierarchical clustering of 8 dpf RNA-Seq samples after removing the batch effect.
(TIF)

S8 Fig High reproducibility of transcriptomic data. Reproducibility of transcriptomic data was analysed using Pearson’s correlation coefficient for 5 dpf (A), as well as for 8 dpf samples before (B) and after (C) removing the batch effect. Pearson’s correlation coefficient values are visualized using heatmaps. Mut–*mia40a* mutant samples; WT- wild-type control samples.
(TIF)

S1 Table Differentially expressed genes and proteins. Data for up- and downregulated genes, as well as proteins in 5 and 8 dpf samples are shown in separate sheets (FDR 5%; log_2_FC = < -0.9 and > = 0.9).
(XLSX)

S2 Table Differentially expressed MitoCarta 2.0 genes at the transcriptomic and proteomic levels. Proteomic and transcriptomic data for 5 and 8 dpf samples are presented in separate sheets.
(XLSX)

S3 Table List of primers used in this study. (XLSX)

S4 Table RNA-Seq results of analysis of *mia40a* mutants (Mut) and wild-type (WT) controls. Expression matrices for 5 and 8 dpf samples are shown in separate sheets.
(XLSX)

S5 Table Expression matrix for 8 dpf RNA-Seq samples after batch effect removal. (XLSX)

S6 Table Quantitative proteomic analysis of *mia40a* mutants (Mut) and wild-type (WT) controls. (XLSX)

S7 Table Quantitative proteomic analysis of *mia40a* mutants (Mut) and wild-type (WT) controls merged on the first entry of semicolon separated gene names. (XLSX)

S8 Table Source data. Source data underlying graphs are shown in separate sheets.
(XLSX)
