# Correction: Loss of the Mia40a oxidoreductase leads to hepato-pancreatic insufficiency in zebrafish

**DOI:** 10.1371/journal.pgen.1007972

**Published:** 2019-01-31

**Authors:** Anna M. Sokol, Barbara Uszczynska-Ratajczak, Michelle M. Collins, Michal Bazala, Ulrike Topf, Pia R. Lundegaard, Sreedevi Sugunan, Stefan Guenther, Carsten Kuenne, Johannes Graumann, Sherine S. L. Chan, Didier Y. R. Stainier, Agnieszka Chacinska

There is information missing from the Funding section. The phrase “National Science Centre grants 2016/23/P/NZ3/03730 (BUR)” should be “POLONEZ Fellowship of National Science Centre, Poland, 2016/23/P/NZ3/03730. This project has received funding from the European Union’s Horizon 2020 research and innovation programme under the Marie Skłodowska-Curie grant agreement No 665778 (BUR).”.
