# Supplementary figures and images for: Loss of the Mia40a oxidoreductase leads to hepato-pancreatic insufficiency in zebrafish
Source: PLoS Genet. 2018 Nov 20;14(11):e1007743. doi: 10.1371/journal.pgen.1007743 (PMC6245507; doi:10.1371/journal.pgen.1007743)

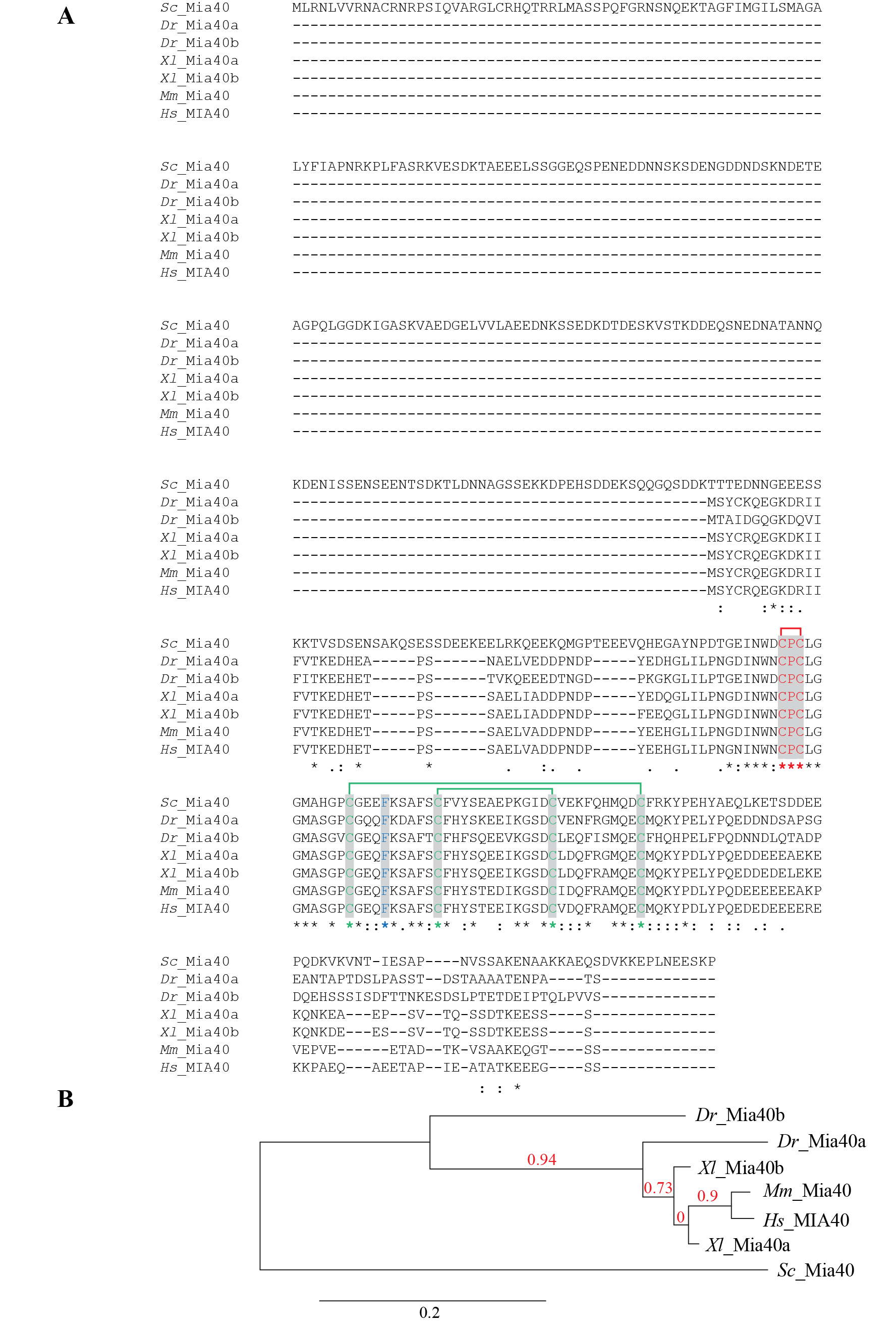

Supplement: S1 Fig — (A) Protein alignment of zebrafish Mia40a and Mia40b with human, mouse, frog and yeast orthologs. Compared to higher eukaryotes, the yeast ortholog contains a large N-terminal presequence and transmembrane domain. The conserved CPC motif is shown in red and the cysteines arranged in the double CX9C motifs in green. The conserved phenylalanine, important for substrate binding, is presented in blue. (B) Phylogenetic analysis of zebrafish Mia40a and Mia40b. (TIF) [file pgen.1007743.s001.tif]

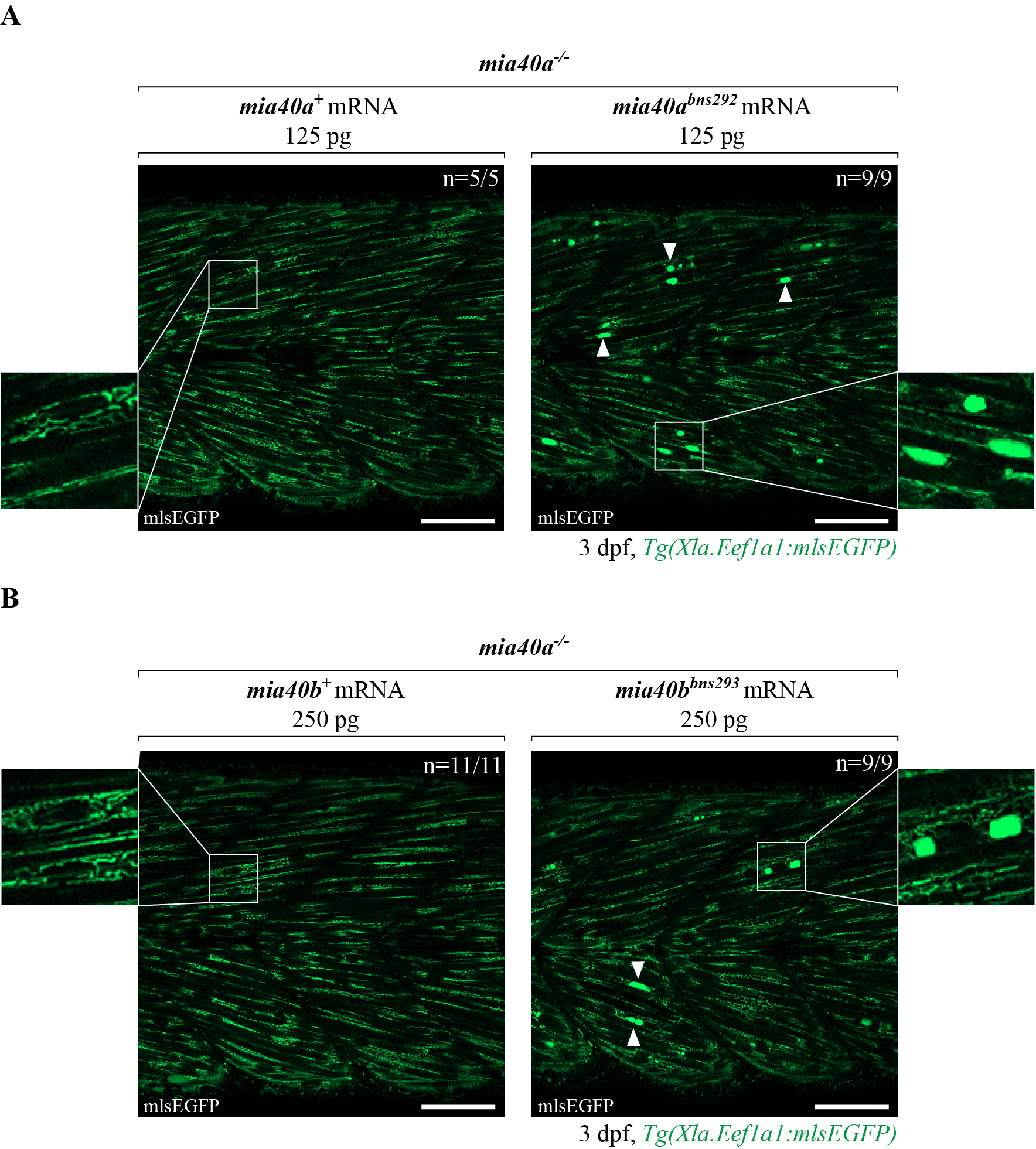

Supplement: S2 Fig — 125 pg of mia40a+ or mia40abns292 mRNA (A) or 250 pg of mia40b+ or mia40abns293 mRNA (B) was injected at 1-cell stage into embryos from a heterozygous in-cross of mia40a+/- in the transgenic background Tg(Xla.Eef1a1:mlsEGFP). The appearance of GFP-positive inclusions in the skeletal muscle of the mia40a-/- larvae at 3 dpf is rescued by injections of the wild-type (A, B, left panels) but not mutant (A, B, right panels) versions of either protein. To better visualize the mitochondrial morphology, a magnified image is presented on the side. Single plane sections are shown. The arrowhead points to a GFP-positive inclusion. Scale bar, 50 μm; n: number of analysed individuals. All images are lateral views, anterior to the left. (TIF) [file pgen.1007743.s002.tif]

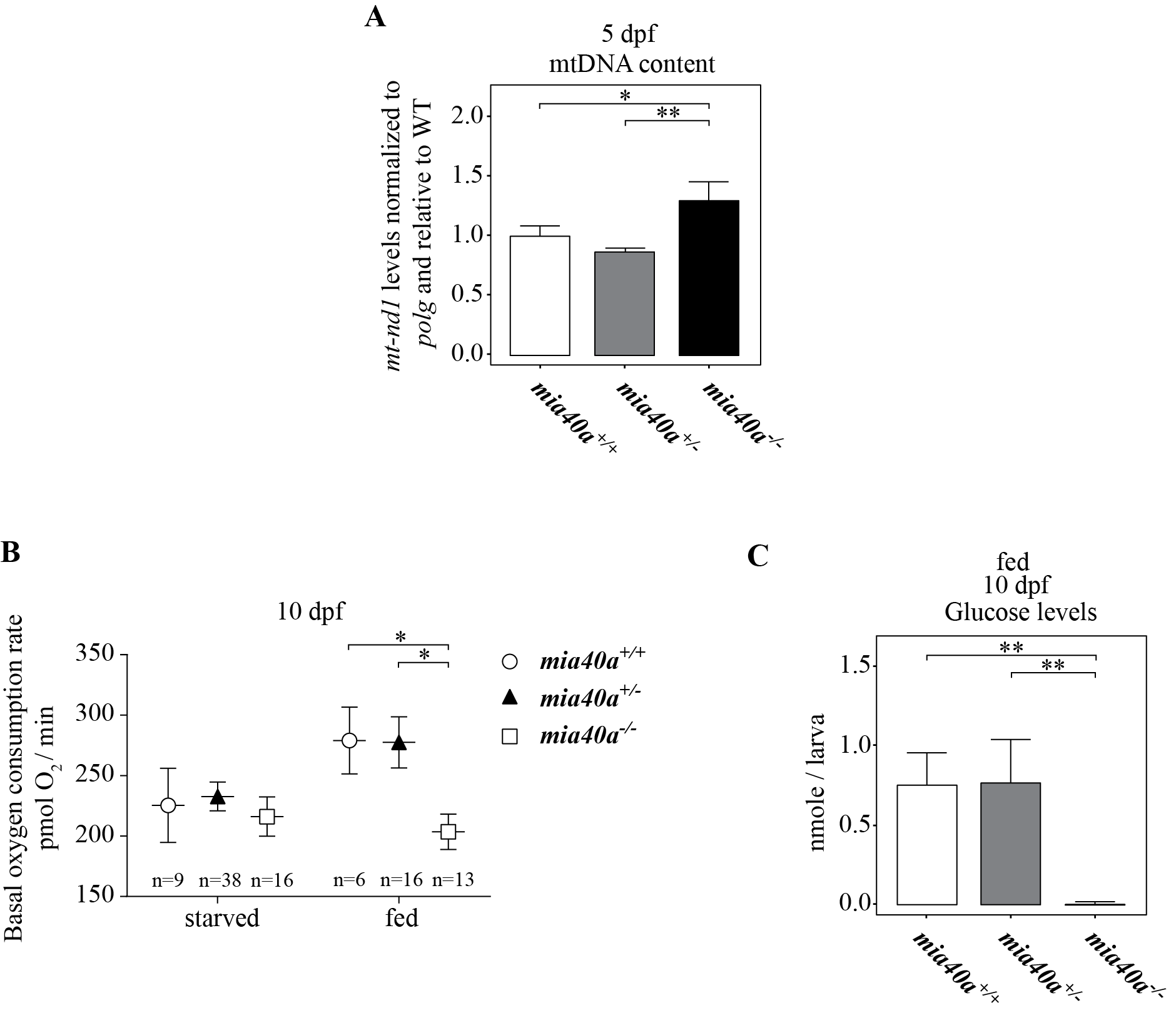

Supplement: S3 Fig — (A) mtDNA copy number was assayed and expressed as the level of the mitochondria-encoded mt-nd1 gene normalized to the nuclear-encoded polg. Larvae with homozygous mutations in the mia40a gene have elevated levels of mtDNA. Data derived from three biological replicates. Error bars correspond to SEM, P < 0.05 (*) and P < 0.01(**) by unpaired t-test. (B-C) Embryos obtained from an in-cross of heterozygous mia40a+/- siblings were continuously fed from 5 dpf or starved. At 10 dpf, the larvae were collected and subjected to respiration analysis using the Seahorse technology (B) or glucose analysis (C). (B) No significant changes in cellular respiration between the compared genotypes are noticed at 10 dpf upon starvation. Nutrient supply results in elevated respiration in the wild-type and heterozygous siblings, but not in mia40a homozygous mutants. Error bars correspond to SEM, P < 0.05 (*) by Mann-Whitney test. (C) Preceding death at 10 dpf, glucose levels in mia40a homozygous mutants are barely detectable. Data derived from three biological replicates. Error bars correspond to SEM, P < 0.01 (**) by unpaired t-test. (TIF) [file pgen.1007743.s003.tif]

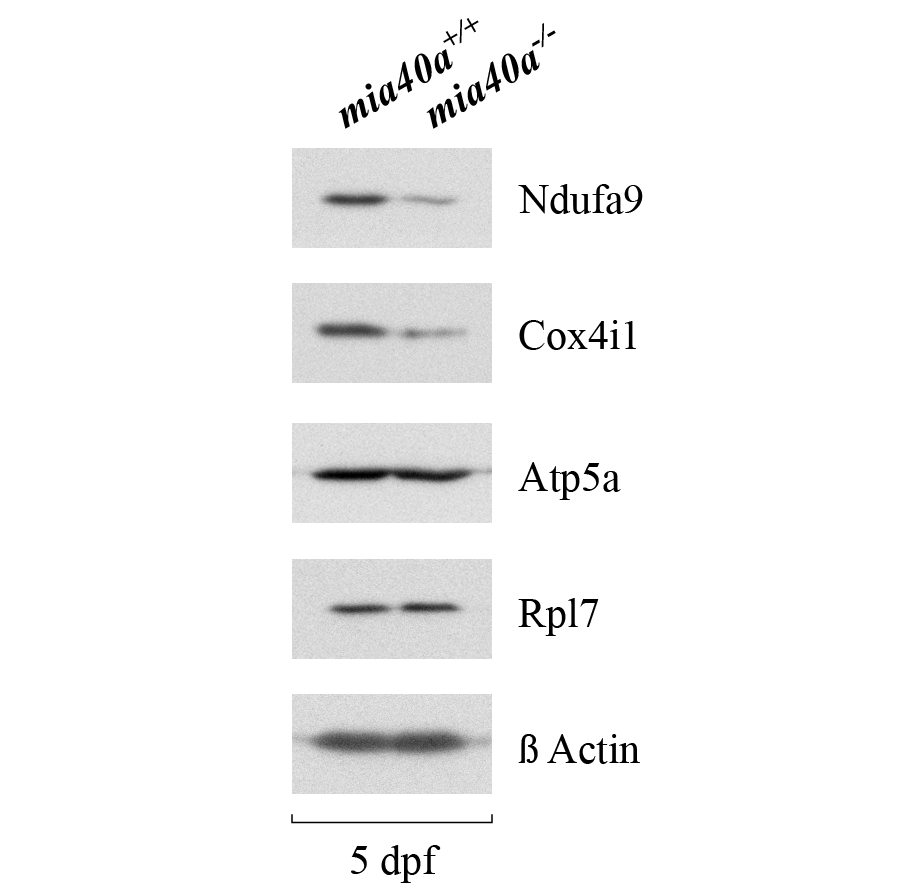

Supplement: S4 Fig — Protein lysates from wild-type or mia40a mutants were subjected to SDS-PAGE analysis and western blotting using specific antibodies against mitochondrial and cytosolic proteins. Representative images for three biological replicates show decreased levels of members of respiratory complex 1 (Ndufa9) and complex 4 (Cox4i1). (TIF) [file pgen.1007743.s004.tif]

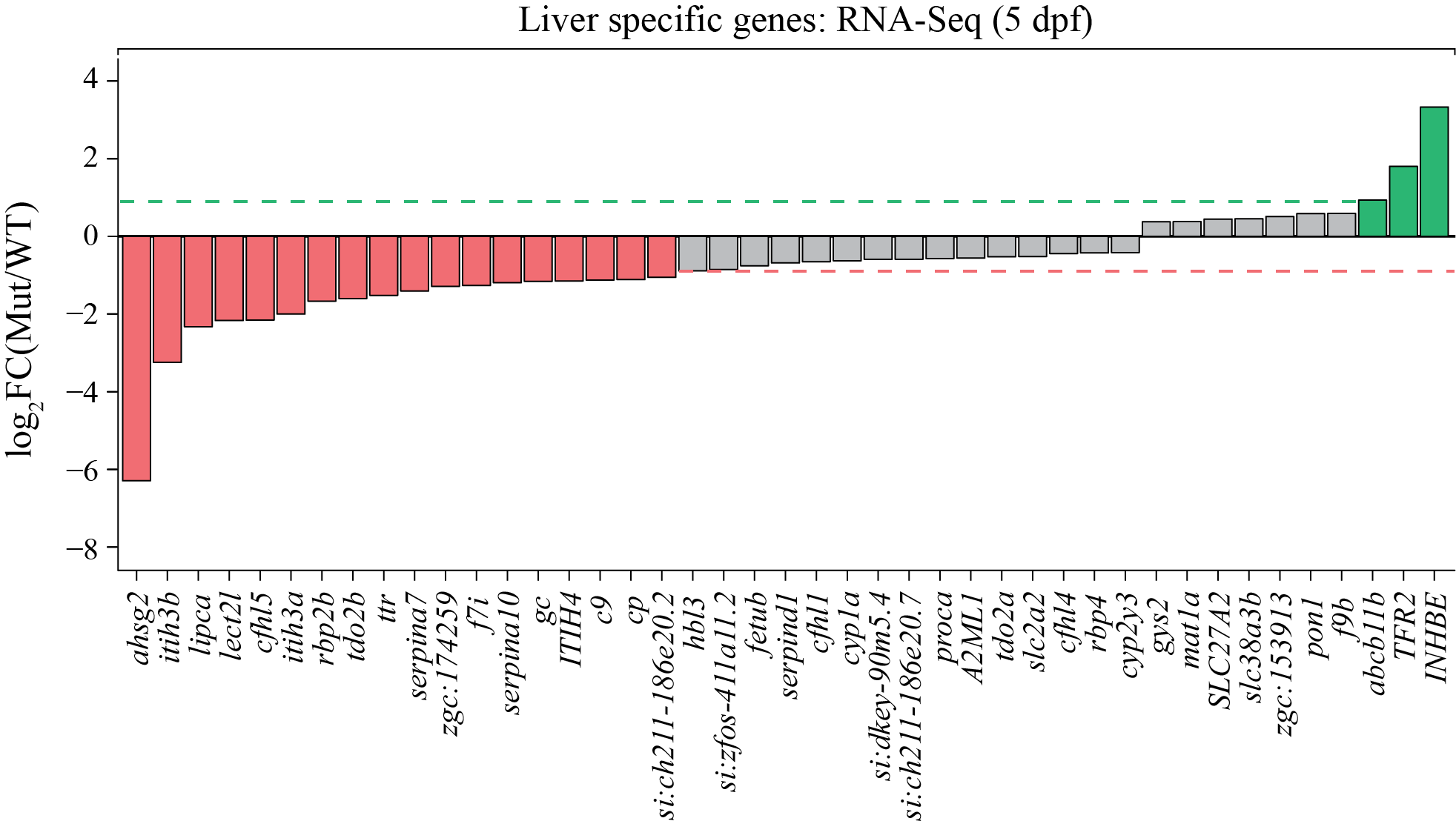

Supplement: S5 Fig — Expression levels of liver specific genes in 5 dpf samples (FDR 5%). The red dashed line represents log2FC = -0.9 and the green dashed line represents log2FC = 0.9. (TIF) [file pgen.1007743.s005.tif]

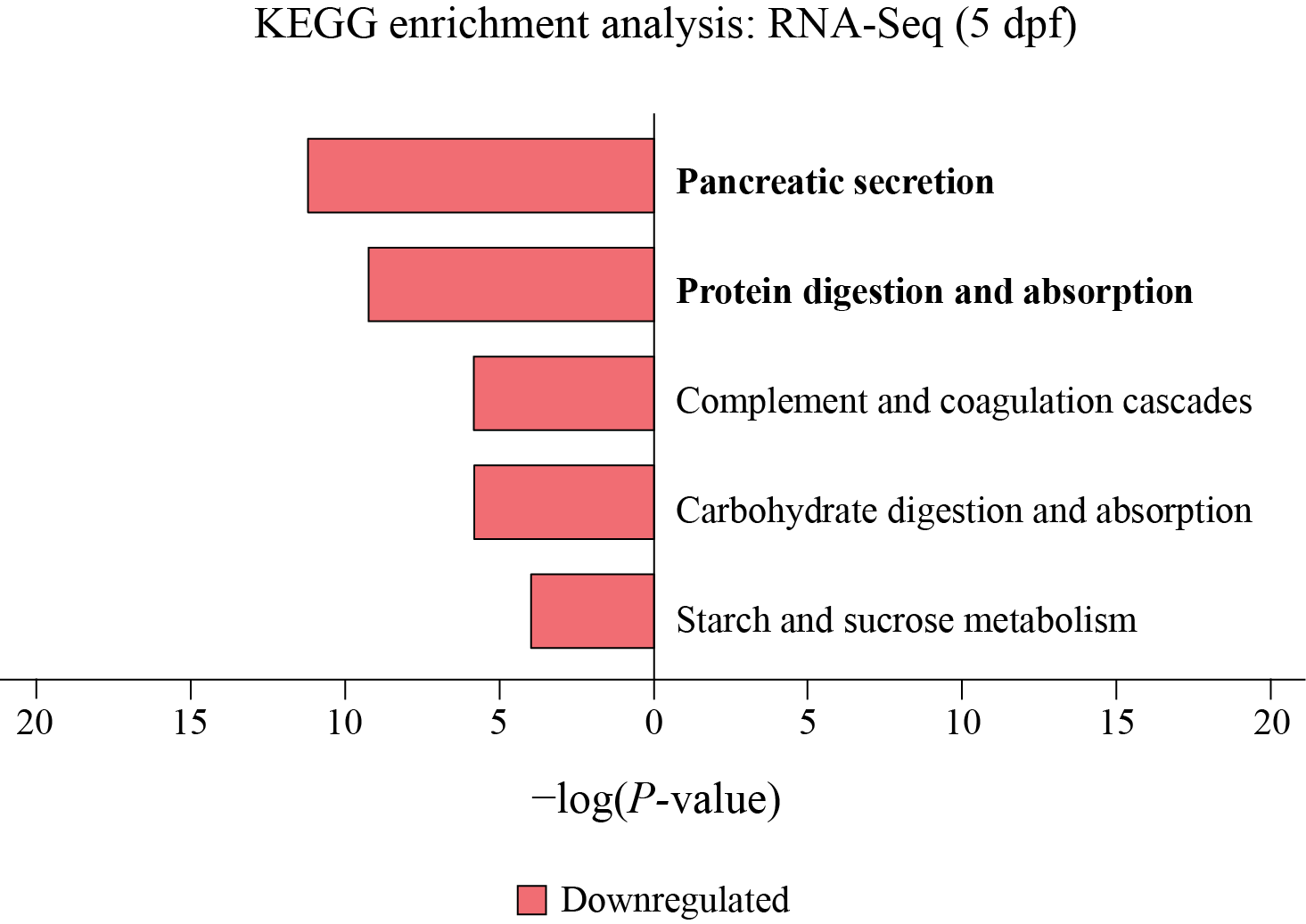

Supplement: S6 Fig — The results are presented as a negative of log10 of P-value after Bonferroni correction. (TIF) [file pgen.1007743.s006.tif]

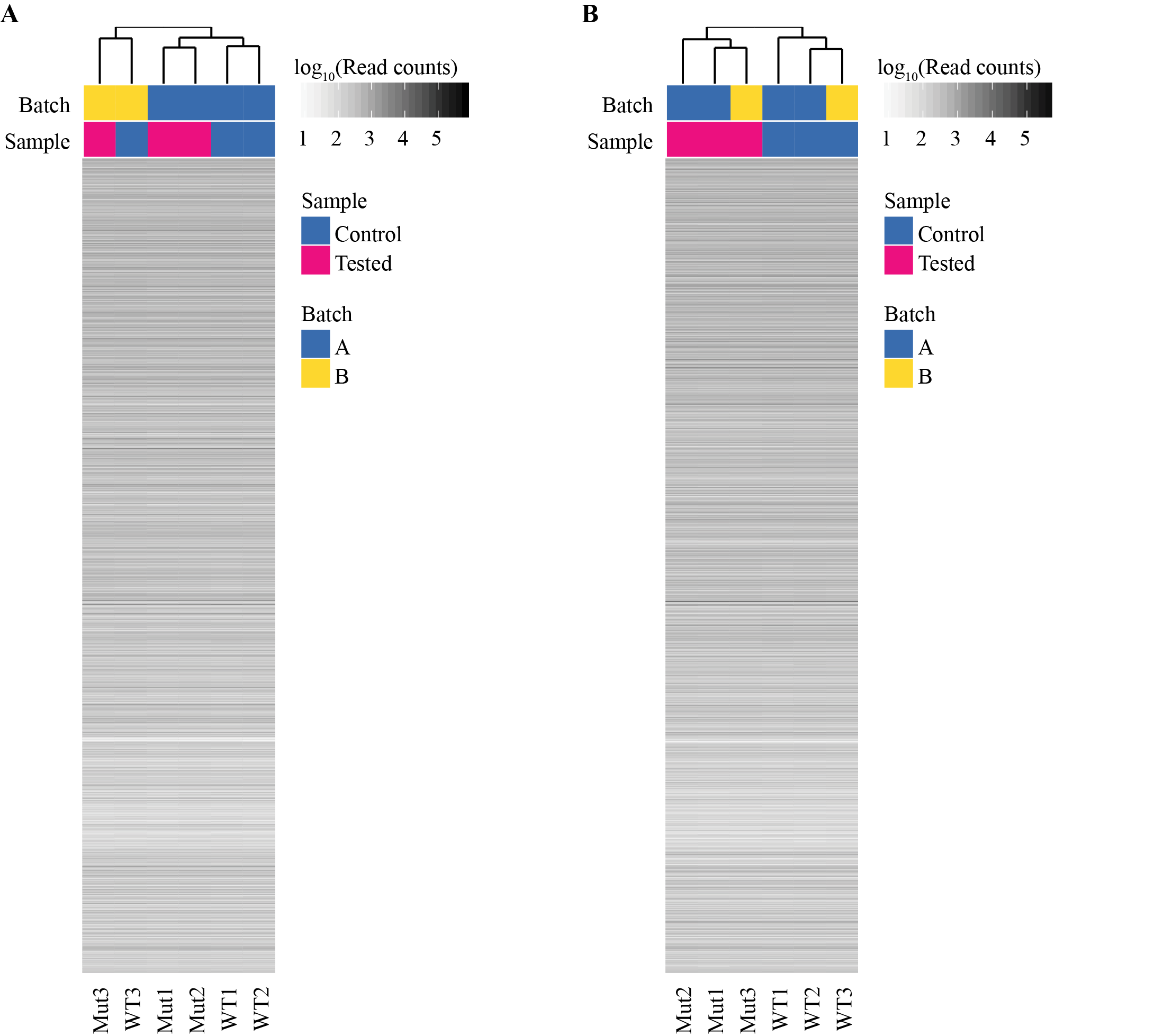

Supplement: S7 Fig — (A) Hierarchical clustering of 8 dpf RNA-Seq samples with batch effect. (B) Hierarchical clustering of 8 dpf RNA-Seq samples after removing the batch effect. (TIF) [file pgen.1007743.s007.tif]

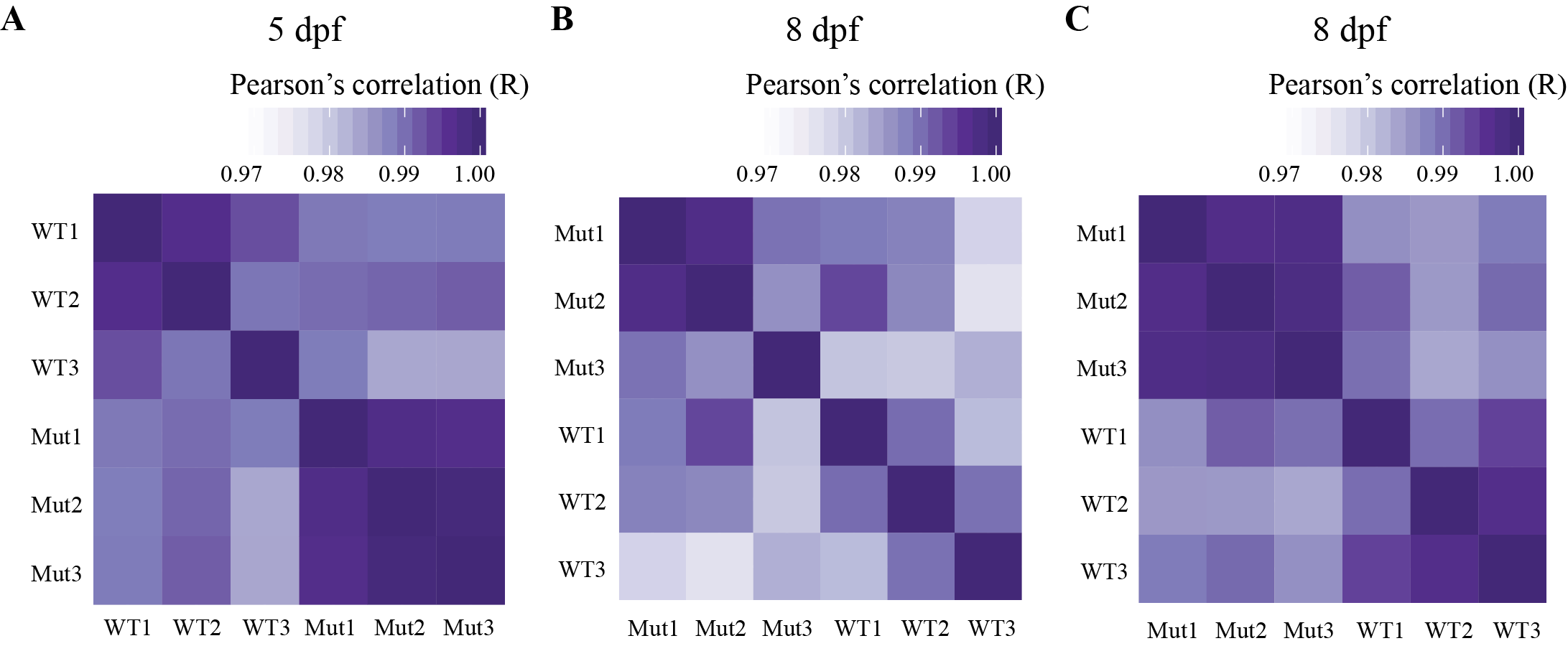

Supplement: S8 Fig — Reproducibility of transcriptomic data was analysed using Pearson’s correlation coefficient for 5 dpf (A), as well as for 8 dpf samples before (B) and after (C) removing the batch effect. Pearson’s correlation coefficient values are visualized using heatmaps. Mut–mia40a mutant samples; WT- wild-type control samples. (TIF) [file pgen.1007743.s008.tif]
